# A synopsis of centipedes in Brazilian caves: hidden species diversity that needs conservation (Myriapoda, Chilopoda)

**DOI:** 10.3897/zookeys.737.20307

**Published:** 2018-02-12

**Authors:** Amazonas Chagas-Jr, Maria Elina Bichuette

**Affiliations:** 1 Departamento de Biologia e Zoologia, Instituto de Biociências, Universidade Federal de Mato Grosso, Avenida Fernando Correa da Costa, 2367, Boa Esperança, 78060–900, Cuiabá, MT, Brasil; 2 Laboratório de Estudos Subterrâneos, Departamento de Ecologia e Biologia Evolutiva, Universidade Federal de São Carlos, Rodovia Washington Luis, Km 235, CP 676, 13565–905 São Carlos, SP, Brasil

**Keywords:** Brazil, cave fauna, Geophilomorpha, Lithobiomorpha, Scolopendromorpha, Scutigeromorpha

## Abstract

This study revises centipede fauna found in Brazilian caves, focusing on troglomorphic taxa and emphasizing conservation status. We present 563 centipede specimens from 274 caves across eleven Brazilian states. Of these, 22 records were derived from existing literature and 252 are newly collected. Specimens represent four orders, ten families, 18 genera, and 47 morphospecies. Together, the cave records represent 21 % of Brazil’s centipede fauna. Scolopendromorpha was the most representative order (41 %), followed by Geophilomorpha (26 %), Scutigeromorpha (23 %), and Lithobiomorpha (10 %). Six species were found only in caves, with four considered troglobitic. The distribution of *Cryptops iporangensis*, the first Brazilian troglobitic centipede species to be discovered, was expanded to other three caves. *Cryptops spelaeoraptor* and *Cryptops iporangensis* are two troglobitic species considered Vulnerable and Endangered, respectively, according to the IUCN Red List. Main threats to Brazilian caves are mining, hydroelectric projects, water pollution, and unregulated tourism.

## Introduction

Centipedes are predatory terrestrial arthropods found in numerous habitats on all continents except Antarctica. More than 3,300 species are distributed across five extant orders and one extinct order. Centipedes are nocturnal and present in diverse microhabitats, including soil, decaying trunks, leaf litter, subterranean galleries, and termite mounds; they have also adapted to widespread environments such as grasslands, deserts, caves, and seashores (Lewis 1981). However, little is known about cave-dwelling centipedes despite how commonly members of these taxa are found in subterranean environments. Within Brazilian fauna alone, cave records exist for four chilopod orders, but very few reports are available regarding obligate cave organisms.

In Europe, approximately 50 troglobitic lithobiomorph species are distributed in caves from Spain, Italy, Romania, and nearly all countries on the Balkan Peninsula (Negrea and Minelli 1994, Culvier and Shear 2012). In Africa, only two cave species have been reported: *Lithobius chickerensis* Verhoeff, 1936 (from Morocco and Algeria) and *Eupolybothrus kahfi* Stoev & Akkari, 2010 (from Tunisia) (Negrea and Minelli 1994, Stoev et al. 2010). Within the troglobitic Geophilomorpha order, the Geophilidae family is represented by two species: *Geophilus persephones* Foddai & Minelli, 1999 from France (Fodаai and Minelli 1999) and *Geophilus hadesi* Stoev, Akkari, Komericki, Edgecombe & Bonato, 2015 from Croatia (Stoev et al. 2015), while the Ballophilidae family is represented by one species: *Ityphilus cavernicolus* Matic, Negrea & Fundora Martinez, 1977 from Cuba (Matic et al. 1977, Lewis 1981). Most troglobitic scolopendromorphs belong to the genera *Cryptops* Leach 1815 (nine species) (Matic et al. 1977, Zapparoli 1990, Negrea and Minelli 1994, Edgecombe 2005, 2006, Ázara and Ferreira 2013, 2014a) and *Newportia* (five species) (Negrea et al. 1973, Chagas-Jr and Shelley 2003, Schileyko 2013, Ázara and Ferreira 2013, 2014b). One *Cryptops* species was recorded in Spain (*C. longicornis* Ribaut, 1915), one in the Canary Islands (*C. vulcanicus* Zapparoli, 1990), one in Italy (*C. umbricus* Verhoeff, 1931), two in Australia (*C. roeplainsensis* Edgecombe, 2005 and *C. camoowealensis* Edgecombe, 2006), and four in the neotropics (*C. cavernicolus* Matic, Negrea & Fundora Martinez, 1977 and *C. troglobius* Matic, Negrea & Fundora Martinez, 1977 from Cuba; *C. iporangensis* Ázara & Ferreira, 2013 and *C. spelaeoraptor* Ázara & Ferreira, 2014 from Brazil). In contrast, *Newportia* is less common, with five currently known troglobitic species: *N. leptotarsis* Negrea, Matic & Fundora-Martinez, 1973 from Cuba; *N. troglobia* Chagas-Jr & Shelley, 2003 from Mexico; *N. stoevi* Schileyko, 2013 from Puerto Rico; as well as *N. spelaea* Ázara & Ferreira, 2014 and *N. potiguar* Ázara & Ferreira, 2014 from Brazil. *Scolopocryptops troglocaudatus* Chagas-Jr & Bichuette, 2015 was also recorded as the first troglobitic species of *Scolopocryptops* Newport, 1845 in Brazil. Finally, *Thalkethops grallatrix* Crabill, 1960 was the only representative of *Thalkethops* Crabill, 1960 found in Carlsbad Cave, New Mexico, USA (Negrea and Minelli 1994).

Cave centipedes (unidentified scutigeromorph and scolopendromorph species) were first recorded in Brazil at the start of the 1980s. These records were mainly from limestone caves in the states of Goiás, Mato Grosso do Sul, Minas Gerais and São Paulo (Dessen et al. 1980, Chaimowicz 1984, Godoy 1986, Trajano 1987, Trajano and Gnaspini-Netto 1991a, b, Gnaspini and Trajano 1994) or from sandstone caves in Pará state (Trajano and Moreira 1991). Unidentified lithobiomorph species have also been observed in limestone caves from southeastern Brazil (Gnaspini-Netto 1989). These records have all been compiled by Pinto-da-Rocha (1995) in one publication on Brazilian cave fauna. Subsequent records include Geophilidae species from the Alto Ribeira karst area, São Paulo state, southeastern Brazil, as well as the first sightings of *Cryptops* and lithobiomorphs from iron caves in Pará and Minas Gerais, respectively (Trajano and Bichuette 2010). A recently published faunistic inventory recorded centipedes from caves in the Serra da Bodoquena karst area, middle Paraguay River basin, in the state of Mato Grosso do Sul (Medeiros et al. 2014). However, these specimens were only tentatively identified to the class level.

Brazil has a high potential for subterranean fauna, with over 15,000 known limestone, sandstone, quartzite, igneous, iron ore, and shale caverns (Canie 2016). However, most caves are not included in conservation units (e.g., parks) or special environmental-protection programs. Despite the large number of potential habitats, relatively few obligate cave-dwelling centipedes are known from Brazil.

Although the description of five troglobitic species within the past ten years has improved our knowledge regarding Brazilian cave centipedes, we still do not fully understand centipede species distribution and the status of their cave habitats. To address this issue, here an annotated list of centipedes from Brazilian caves is presented, including distribution data, taxonomical notes, and considerations for conservation.

## Materials and methods

Data were obtained from two sources: (1) literature searchers and (2) scientific collections at the following locations:

**MNRJ** Museu Nacional, Universidade Federal do Rio de Janeiro, Rio de Janeiro, Brazil;

**MZUSP** Museu de Zoologia da Universidade de São Paulo, São Paulo, Brazil;

**UFSCAR** Universidade Federal de São Carlos, São Paulo, Brazil;

**UFMT** Universidade Federal de Mato Grosso, Cuiabá, Mato Grosso, Brazil.

Of the 563 recorded specimens, 267 were identified at species level (seven specimens from the literature), 221 at genus level, 53 at family level, and 22 at order level. Most specimens from scientific collections (296) did not allow precise identification, but were still useful as indicators of species distribution and guides for future collection. This high number of unidentified species was due to two major reasons: 1) inadequate conservation in ethanol (resulting in damage such as lacking the ultimate pair of legs); 2) the danger of desiccation precluding analysis of intra– and interspecific variation among existing specimens, given the lack of back-up samples. Materials were identified through visual inspection in the field, then captured with forceps and brushes, without the aid of traps or attractive baits. Part of the material comes from the authors’ collections and donations from environmental consulting firms.

Geographic coordinates were taken from specimen labels and the database of the Laboratório de Estudos Subterrâneos (Universidade Federal de São Carlos). Maps were prepared in Qgis 2.18 (QGIS 2017).

Subsequent descriptions of each taxon include the following information: published records, material examined with repositories, specimen count, localities, collection date and collector/s (when available), taxonomic notes, distribution, habitat/microhabitat (related to the cave records), as well as conservation considerations, when appropriate. Furthermore, vegetation type, cave lithology, geomorphological group, and/or karst area are presented per species/morphospecies, in addition to being recorded on the map.

When possible, the centipedes were classified according to the three ecological-evolutionary categories of cave fauna: troglobites (obligatory subterranean populations); troglophiles (populations well established in caves and epigean habitats, with individuals regularly commuting between the two), and trogloxenes (populations inhabit caves but leave to complete life cycles) (Barr 1968). Troglobites generally present troglomorphisms (characteristics linked to life in permanent darkness), with the most common being reduction (or lack) of eyes and body pigmentation (Christiansen 1962, Trajano 1993). “Accidentals” are individuals that can survive temporarily in caves but are not considered a cave faunal category.

## Results

**Figure 1.**
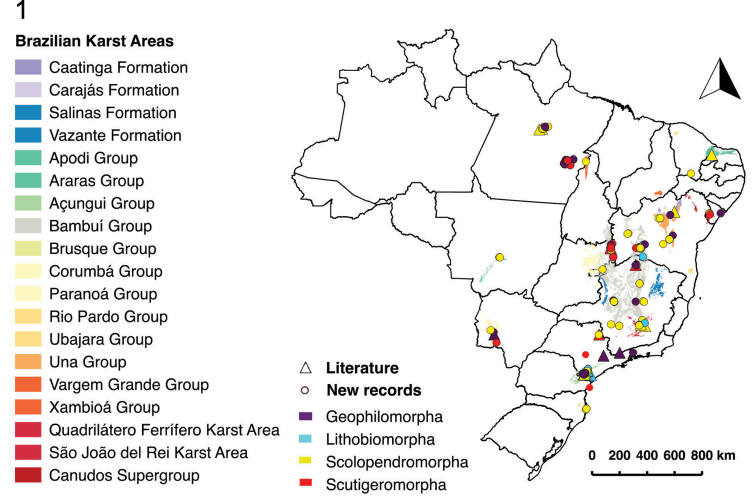
Map of Brazil showing the distribution of centipedes in different lithologies and geomorphological groups.

**Figure 2.**
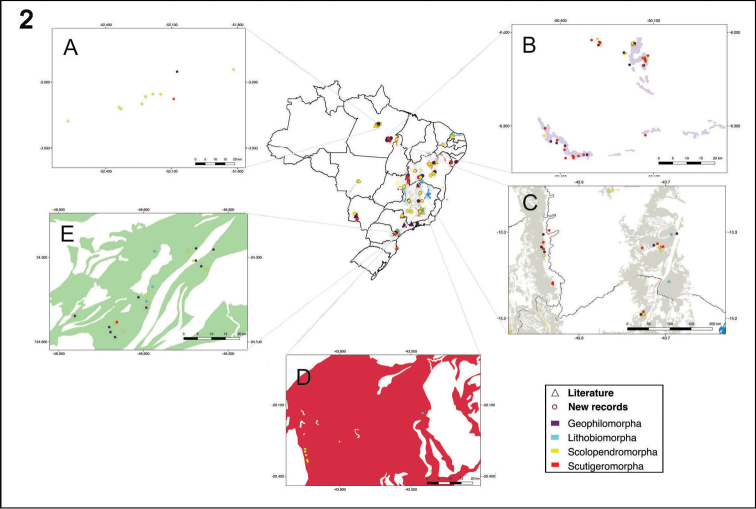
Map of Brazil showing the distribution of centipedes per regions. **A** Secondary map of Pará state: Altamira region **B** Secondary map of Pará state: Parauapebas and Canaã dos Carajás region **C** Secondary map of Goiás and Bahia states **D** Secondary map of Minas Gerais state **E** Secondary map of São Paulo state.

Centipedes were recorded in 274 caves across eleven states (Figures 1 and 2). Analyzed data include 22 records from the literature and 252 new samples (from scientific collections, monitoring projects, and donated material).

### Class Chilopoda

#### Order Scutigeromorpha Pocock, 1895

##### Family Pselliodidae Chamberlin, 1921

###### Genus *Sphendononema* Verhoeff, 1904

####### Sphendononema guildingii

Taxon classificationAnimaliaScutigeromorphaPselliodidae

(Newport, 1845)

######## Published records.

None.

######## Material examined.

**PARÁ**: Parauapebas (Equatorial Rainforest and “Campos Rupestres”), Iron Ore: Gruta N5E 02, Gruta N5E 09, Gruta N5S 20, (MNRJ) 4 spec, 22.iii–03.iv.2005, Andrade, R. & Arnone, IS; Gruta N5S 02, Gruta N5S 06, (MNRJ) 4 spec, 03–13.v.2005, Andrade, R. & Arnone, I; Gruta S11C 14, Gruta S11D 96, Gruta S11D 01, Gruta S11B 49, Gruta S11B 23, Gruta S11D 12, Gruta S11B 13, Gruta S11A 26, Gruta S11A 26, Gruta S11C 08, Gruta S11D 39, Gruta S11D 55, Gruta S11 D10, (MNRJ) 26 spec, 23.viii–02.ix.2007, Andrade, R.; Gruta N1 22, Gruta N1 37, Gruta N1 173, Gruta N1 25, Gruta N1 37, (MNRJ) 11 spec, 28.ix–03.x.2007, Andrade, R.; Gruta N5S 37 CL; Gruta N4E 14 AF; Gruta N4E 10AF; Gruta N5S 8AF; Gruta N5S 5 8AF; Gruta N5S 21AF; Gruta N4E 26AF; Gruta N4E 61CL; Gruta N4E 26 CL; Gruta N4E 61 AF; Gruta N5S 8 CL; (MNRJ) 18 spec, 07–12.x.2008, Andrade, R. et al.; Gruta GEM 1578 CL, (MNRJ) 3 spec, 17–24.x.2008; Curionópolis (Equatorial Rainforest and “Campos Rupestres”), Iron Ore: Gruta SL 74 CL, (MNRJ) 4 spec, 17–24.x.2008, Andrade, R. et al.; Canaã dos Carajás (Equatorial Rainforest and “Campos Rupestres”), Iron Ore: Gruta NV 06, (MNRJ) 1 spec, 22–28.ii.2005, Andrade, R., Arnone, I.; Gruta S11C 0124, Gruta S11C 0147, Gruta S11C 0153, (UFMT) 4 spec, 01–08.viii.2015, BioEspeleo Consultoria Ambiental; Caverna ST 0068, (UFMT) 1 spec, 26.i.2016, BioEspeleo Consultoria Ambiental; Altamira (Equatorial Rainforest), Sandstone and Shale: Caverna Leonardo da Vinci, (UFSCAR) 1 spec, xii.2010, Bichuette, ME., Gallão, JE., von Schimonsky, DM.; Abrigo Paratizinho, (UFSCAR) 2 spec, 04.iv.2011, Bichuette, ME., Gallão, JE., Pedroso, DR., von Schimonsky, DM. **GOIÁS**: São Domingos (Cerrado), Limestone: Lapa São Mateus-Imbira III, (UFSCAR) 1 spec, 11.vii.1988, Gregeo UnB, Vendramini, G.; (UFSCAR) 1 spec, 13.ii.2012, Bichuette, ME.; Lapa Angélica, (UFSCAR) 1 spec, 20.iv.2011 and (UFSCAR) 1 spec, 21.iv.2011, Bichuette, ME.; (UFSCAR) 1 spec, 31.x.2012, Bichuette, ME.; Caverna Sumidouro I, (UFSCAR) 1 spec, 25.iv.2012 and (UFSCAR) 1 spec, 17.vi.2012, von Schimonsky, DM., Gallão, JE., Fernandes, CS; Formosa (Cerrado), Limestone: Buraco das Araras, (UFSCAR) 1 spec, 06.x.2011, Vidal, G.M.; Mambaí (Cerrado), Limestone: Gruta Judite, (UFSCAR) 2 spec, 01.v.2013, Bichuette, ME., Gallão, JE., von Schimonsky, DM., Rizzato, PP., Borghezan, R.; Gruta Pasto de Vacas, (UFSCAR) 1 spec, 02.v.2013, Bichuette, ME., Gallão, JE., von Schimonsky, DM., Rizzato, PP., Borghezan, R.; **MATO GROSSO**: Nobres (Cerrado), Limestone: Toca da Serra Rica, (UFSCAR) 3 spec, 04.v.2015, Bichuette, ME., Chagas-Jr, A., Nunes, GA.; Toca do Sorvete, (UFSCAR) 1 spec, 06.v.2015, Bichuette, ME., Chagas-Jr, A., Nunes, GA.; **Mato Grosso DO SUL**: Bonito (Cerrado), Limestone: Gruta São Mateus, (UFSCAR) 1 spec, 26.vi.2012, Cordeiro, LM., Borghezan, R.; **SERGIPE**: Japaratuba (“Restinga” – Atlantic Forest and coastal vegetation), Limestone: Caverna Casa do Caboclo, (UFSCAR) 2 spec, 19.x.2014, Bichuette, ME.; Simão Dias (Caatinga), Limestone: Toca da Raposa, (UFSCAR) 2 spec, 19.x.2014, Bichuette, ME.; **BAHIA**: Carinhanha (Caatinga), Limestone: Gruna das Três Cobras, (UFSCAR) 1 spec, 10.ix.2008, Bichuette, ME.; Gruna Google, (UFSCAR) 2 spec, 13.ix.2008, Bichuette, ME.; Gruna Vila Nova, (UFSCAR) 1 spec, 26.vii.2012, Bichuette, ME., Gallão, JE., Rizzato, PP.; Gruna das Três Cobras, (UFSCAR) 1 spec, 30.v.2012, Bichuette, ME., Gallão, JE.; Caverna Bem Bom, (UFSCAR) 1 spec, 28.xi.2015, Gallão, JE., Bichuette, ME.; Andaraí (“Campos rupestres” highland heterogeneous vegetation on rocks), Sandstone: Gruna dos Torras, (UFSCAR) 1 spec, 19.i.2007, Bichuette, ME.; Coribe (Caatinga), Limestone: Gruna do Enfurnado, (UFSCAR) 2 spec, ix.2007, Trajano, E., Sansone, D.; **MINAS GERAIS**: Januária (Cerrado-Caatinga transition), Limestone: Gruta do Janelão, (UFSCAR) 5 spec, 22.vii.2012, Bichuette, ME.; Itacarambi (Cerrado-Caatinga transition), Limestone: Lapa do Branco I, (UFSCAR) 1 spec, vi.2014 by Monte, BGO.; Lapa do Cipó, (UFSCAR) 1 spec, 10.iv.2015, Gallão, JE., Monte, BGO and (UFSCAR) 1 spec, 26.viii.2014, Bolfarini, MP., Monte, BGO.; Caverna Troncos, (UFSCAR) 1 spec, 11.iv.2015, Gallão, JE., Monte, BGO.; **SÃO PAULO**: Altinópolis (Cerrado and Semidecidous seasonal forest), Sandstone: Gruta Itambé, (UFSCAR) 1 spec, 30.vi.2011, Bichuette, ME.; Caverna Olho de Cabra, (UFSCAR) 1 spec, 23.viii.2016, Gallo, JS., Mello, RV, Ferro, JP.

######## Taxonomic notes.

*Sphendononema guildingii* is among the most common centipedes in Brazilian caves. Two specimens from Caverna Casa do Caboclo in Sergipe state, northeastern Brazil exhibit different tergal pigmentation patterns and might not be conspecifics of *S. guildingii*.

######## Distribution.

This species is widespread in Brazil, found in almost all states, except Paraná, Santa Catarina, and Rio Grande do Sul. Classified as a troglophile, *S. guildingii* was observed in limestone, quartzitic, sandstone, and iron ore caves.

######## Habitat.

Cave (rocky substrate).

######## Conservation.

Despite its wide distribution, the species is under threat because mining and/or hydroelectrical projects are affecting numerous cave habitats (e.g., in Pará and Minas Gerais states). Combined with their low-medium abundance and the lack of ecological or molecular studies, protection for this species should be considered.

##### Family Scutigeridae Gervais, 1847

###### Genus *Thereuoquima* Bücherl, 1949

####### Thereuoquima admirabilis

Taxon classificationAnimaliaScutigeromorphaScutigeridae

Bücherl, 1949

######## Published records.

None.

######## Material examined.

**SÃO PAULO**: Iporanga (Atlantic Rainforest): Caverna Santana, (UFSCAR) 1 spec, 16–20.ix.2011, Pellegatti-Franco, F.; **SANTA CATARINA**: Florianópolis (“Restinga” – Atlantic Forest and coastal vegetation), Granite: Caverna da Água Corrente, (UFSCAR) 2 spec, 29–30.ix.2016, Gallão, JE., Xavier, P.; Caverna da Água Corrente, (UFSCAR) 1 spec, 01.v.2016, Bichuette, ME., Gallão, JE., Lee, D., Xavier, P.

######## Distribution.

*Thereuoquima* is a monotypic genus exclusive to Brazil described from Ilha da Queimada Pequena island in São Paulo (Bücherl 1949). Compared with *S. guildingii*, *T. admirabilis* is rarely found outside caves, suggesting that it is troglophilic. Its range is restricted to São Paulo, Paraná, and Santa Catarina, indicating a more temperate distribution. This study presents the first record of *Thereuoquima admirabilis* in limestone and granitic caves from São Paulo and Santa Catarina (south Brazil), respectively.

######## Habitat.

Rocky and unconsolidated substrate.

######## Conservation.

The species’ restricted distribution must be considered for conservation purposes; caves from Santa Catarina are not legally protected and urban expansion is a major threat.

#### Order Lithobiomorpha

##### Family Henicopidae

###### Genus *Lamyctes* Meinnert, 1868

####### Lamyctes spp.

Taxon classificationAnimaliaLithobiomorphaHenicopidae

######## Material examined.

**PARÁ**: Curionópolis (Equatorial Rainforest and “Campos Rupestres”), Iron Ore: Gruta SL 89 CL, Gruta SL 74 CL, (MNRJ) 2 spec, 17–24.x.2008, Andrade, R.; **MATO GROSSO**: Nobres (Cerrado), Limestone: Toca do Sorvete, (UFSCAR) 1 spec, 06.v.2015, Bichuette, ME., Chagas-Jr, A., Nunes, GA.; **BAHIA**: Andaraí (“Campos rupestres” highland heterogeneous vegetation on rocks), Sandstone: Gruna Canal da Fumaça, (UFSCAR) 1 spec, 14.iv.2014, Gallão, JE.; Iuiu (Caatinga): Lapa do Baixão, (UFSCAR) 1 spec, 17.x.2014, Gallão, JE., von Schimonsky, DM.; Carinhanha (Caatinga), Limestone: Caverna Bem Bom, (UFSCAR) 1 spec, 28.xi.2015, Gallão, JE., Bichuette, ME.; **MINAS GERAIS**: Presidente Olegário (Cerrado and Semideciduous seasonal forest), Limestone: Lapa Zé de Sidinei, (UFSCAR) 1 spec, 14.vi.2014, (UFSCAR) 1 spec, 20.i.2014, and (UFSCAR) 3 spec, 24.i.2014, Zepon, T., Resende, LPA., (UFSCAR) 1 spec, 09.ix.2014, Zepon, T., (UFSCAR) 1 spec, 16.iv.2014, Zepon, T., Resende, LPA., Damasceno, GF.; Lapa do Moacir, (UFSCAR) 1 spec, 17.iv.2014, Zepon, T., Resende, LPA., (UFSCAR) 1 spec, 20.i.2014, Zepon, T., Resende, LPA.; Itabirito (“Canga”– heterogeneous flora), Iron Ore: Gruta VL 03 Mina Várzea do Lopes, (MNRJ) 1 spec, 18–25.iv.2007, Andrade, R.; Mariana (“Campos rupestres”): Gruta do Centenário, (UFSCAR) 1 spec, 11.viii.2012, Senna-Horta, L.; **SÃO PAULO**: Apiaí/ Iporanga (Atlantic Rainforest), Limestone: Gruta do Minotauro, (UFSCAR) 1 spec, 14–16.ix. 2009, Pellegatti-Franco, F.; Gruta da Santa, (UFSCAR) 1 spec, 26–30.iii.2009, Pellegatti-Franco, F.; Arapeí (Atlantic Rainforest): Gruta Rio do Capitão Mor 1, (UFSCAR) 1 spec, 23.ii.2013, Gallão, JE.; Ribeirão Grande (Atlantic Rainforest), Limestone: Caverna Cherol, (UFSCAR) 1 spec, 09.vii.2006, Andrade, R., Arnone, IS.; Caverna Cherol, (UFSCAR) 1 spec, 15.iv.2014, Bolfarini, MP.; Caverna Córrego Principal, (UFSCAR) 1 spec, 09.xi.2014, Bolfarini, MP.; Caverna Felício, (UFSCAR) 3 spec, 15.v.2015, Bolfarini, MP.; Iporanga (Atlantic Rainforest), Limestone: Gruta da Água Suja, (UFSCAR) 2 spec, 13–20.iv.2009, Pellegatti-Franco, F.; Gruta Areias de Cima, (UFSCAR) 1 spec, x.2003 and (UFSCAR) 1 spec, xii.2003 by Bessi, R.; (UFSCAR) 1 spec, 30.iv.1990, Trajano, E.; Gruta Casa de Pedra, (UFSCAR) 1 spec, 21.iv.1991 and (UFSCAR) 3 spec, 29.iv.1990, Trajano, E.; Caverna Alambari de Baixo, (UFSCAR) 1 spec, 22.iii.1986, Trajano, E.; Caverna Temimina, (UFSCAR) 1 spec, 26.ix.1988, Trajano, E.; Caverna Betari de Baixo, (UFSCAR) 1 spec, 23.ix.1989, Trajano, E.; Caverna Arataca, (UFSCAR) 1 spec, 28.iii.1991 by Trajano, E.; Caverna Laje Branca, (UFSCAR) 1 spec, 02.viii.2013 Bichuette, ME.; Caverna Passoca de Baixo, (UFSCAR) 1 spec, 03.viii.2013 Bichuette, ME.

######## Taxonomic notes.

Little is known about Brazilian lithobiomorph taxonomy. All specimens examined here belong to *Lamyctes* (Henicopidae), a diverse genus with around 42 species distributed worldwide. In Brazil, only two species have been observed: *L. adisi* Zalesskaja 1994 and *L. emarginatus* (Newport, 1844). The former is endemic to Manaus and only found in Tarumã-Mirim Igapó of the Rio Negro region (Adis 1992, Zalesskaja 1994, Foddai et al. 2002). The latter is an exotic species, now widely distributed in Brazil in addition to being known from Europe, North America, Greenland, Tasmania, and New Zealand.

######## Distribution.

Only a few records of lithobiomorphs are available for Brazil in the literature, mainly stemming from the Amazonian and Atlantic Forests in the southeast. Bücherl (1942) described sightings of *Lithobius forficatus* (Linnaeus, 1758) in Bahia state. This species was originally identified by Brölemann (1909). However, Bücherl (1942) suggests that the specimen in that publication was not *L. forficatus*, but an unknown introduced lithobiomorph. Here, we recorded *Lamyctes* specimens in several caves from Pará, Mato Grosso, Bahia, Minas Gerais, and São Paulo.

######## Habitat.

The genus appeared common in caves of different lithologic characteristics, including limestone, quartzitic, iron, and sandstone, but was preferentially found in limestone caves; no sightings have been recorded for granitic caves. This preference is corroborated by additional records of unidentified lithobiomorphs in limestone caves from Minas Gerais and São Paulo in southeastern Brazil (Gnaspini-Netto 1989, Gnaspini and Trajano 1994, Trajano 1987, Trajano and Gnaspini-Netto 1991a, Pinto-da-Rocha 1995). Certain substrates also seem to be preferred, specifically guano piles, wet clay, or wet vegetal debris. This observation suggests that the genus either favors wet organic substrates (possibly because their potential prey also occurs in such habitats) or is intolerant to dry substrates.

#### Order Scolopendromorpha Pocock, 1895

##### Family Scolopendridae Newport, 1844

###### Subfamily Scolopendrinae Newport, 1844

####### Genus *Cormocephalus* Newport, 1844

######## Cormocephalus impressus

Taxon classificationAnimaliaScolopendromorphaScolopendridae

Porat, 1876

######### Published records.

None.

######### Material examined.

**PARÁ**: Altamira (Equatorial Rainforest), Sandstone: Abrigo Assurini, (UFSCAR) 1 spec, xii.2010, Bichuette, ME., Gallão, JE., von Schimonsky, DM.

######### Distribution.

*Cormocephalus impressus* is distributed in the Antilles, Peru, Ecuador, and Brazil (Bücherl 1942, 1974). This species has been observed in northern, central, and western Brazil, but a unique specimen from one cave in the Amazonian region strongly suggests that *C. impressus* is an accidental species in Brazilian caves.

######### Habitat.

Cave (unconsolidated substrate – sand).

######### Conservation.

*Cormocephalus impressus* is under threat locally due to the construction of a huge hydroelectrical dam in the Altamira region (Belo Monte) that will flood Abrigo Assurini (M.E. Bichuette, pers. comm.).

####### Genus *Rhoda* Meinert, 1886

######## Rhoda thayeri

Taxon classificationAnimaliaScolopendromorphaScolopendridae

Meinert, 1886

######### Published records.

None.

######### Material examined.

**PARÁ**: Parauapebas (Equatorial Rainforest and “Campos Rupestres”), Iron Ore: Gruta N4E 16, (MZUSP) 1 spec, 20.x–01.xi.2006, Andrade, R.

######### Distribution.

This species is only known from Belém, Pará, and here we provide the first record of its occurrence in an iron ore cave of Parauapebas. Mining projects are extremely frequent in this region. Accidental in caves.

######### Habitat.

Unknown.

####### Genus *Scolopendropsis* Brandt, 1841

######## Scolopendropsis bahiensis

Taxon classificationAnimaliaScolopendromorphaScolopendridae

(Brandt, 1841)

######### Published records.

None.

######### Material examined.

**MINAS GERAIS**: Diamantina (“Campos rupestres” highland heterogeneous vegetation on rocks), Quartzite: Lapa dos Pombos, (UFSCAR) 1 spec, 06.ix.2013, Fonseca-Ferreira, R.

######### Distribution.

A species exclusive to Brazil, *S. bahiensis* is usually found in the semi-arid regions of Bahia and Minas Gerais. This study recorded its occurrence for the first time in a quartzitic cave from Minas Gerais. Accidental in caves.

######### Habitat.

Cave (unconsolidated substrate, under rocks).

######## Scolopendropsis

Taxon classificationAnimaliaScolopendromorphaScolopendridae

sp.

######### Material examined.

**PARÁ**: Canaã dos Carajás (Equatorial Rainforest and “Campos Rupestres”), Iron Ore: Caverna ST 0022, (UFMT) 1 spec, 02.ii.2016, BioEspeleo Consultoria Ambiental.

######### Distribution.

The species was found only in a single iron ore cave from Pará. Accidental in caves.

######### Habitat.

Unknown.

######### Conservation.

Due to heavy impact from mining, the region must be considered in conservation projects. As the species appears to be novel, more samples are needed for detailed taxonomical studies.

####### Genus *Scolopendra* Linnaeus, 1758

######## Scolopendra viridicornis

Taxon classificationAnimaliaScolopendromorphaScolopendridae

Newport, 1844

######### Published records.

None.

######### Material examined.

**GOIÁS**: São Domingos (Cerrado), Limestone: Caverna Bezerra, (UFSCAR) 1 spec, 19.vi.2012, Bichuette, ME.; **BAHIA**: São Desidério (Cerrado-Caatinga transition), Limestone: Gruta do Catão, (UFSCAR) 1 spec, 03.xi.2012, Bichuette, ME.; Paripiranga (Caatinga), Limestone: Caverna Furna do Fim do Morro do Parafuso, (UFSCAR) 1 spec, ix.2013, Rocha, KG.; **MINAS GERAIS**: Itacarambi (Cerrado-Caatinga transition), Limestone: Gruta Olhas d’ Água, (UFSCAR) 1 spec, 23.x.2013, Bichuette, ME.

######### Distribution.

A widespread species in Brazil (Bücherl 1942), *S. viridicornis* is more common in the north, northeastern, central, western, and southeastern regions. The four specimens recorded here were from limestone caves; no data exist regarding its occurrence in other lithologies. Accidental in caves.

######### Habitat.

Cave (rocky substrate, under rocks and trunks).

###### Subfamily Otostigminae

####### Genus *Otostigmus* Porat, 1876

######## Subgenus Dactylotergitius Verhoeff, 1937

######### Otostigmus (Dactylotergitius) caudatus

Taxon classificationAnimaliaScolopendromorphaScolopendridae

Brölemann, 1902

########## Published records.

None.

########## Material examined.

**PARÁ**: Altamira (Equatorial Rainforest), Sandstone: Abrigo da Gravura, (UFSCAR) 1 spec, 08.vii.2009, Bichuette, ME.; Nanoabrigo, (UFSCAR) 1 spec, 13.v.2011 by Gallão, JE.; **SÃO PAULO**: Iporanga (Atlantic Rainforest), Limestone: Gruta dos Paiva, (UFSCAR) 1 spec, 02.iii.2014, Bichuette, ME., Gallão, JE.

########## Distribution.

This species is widely distributed from Brazil to northeast Argentina (Chagas-Jr et al. 2007). Though more common in southeast Brazil, it has also been found in the central, northeastern, and northern regions. Our records place the species in sandstone caves from Pará and in one limestone cave from São Paulo. Accidental in caves.

########## Habitat.

Cave (under rocks).

########## Conservation.

Even with these few records, the wide distribution suggests that this species is not under threat. However, their range in Pará is located within the boundaries of a large hydroelectrical dam (Belo Monte) (M.E. Bichuette, per. obs.), suggesting the possibility of a local threat.

######## Subgenus Parotostigmus Pocok, 1896

######### Otostigmus (Parotostigmus) amazonae

Taxon classificationAnimaliaScolopendromorphaScolopendridae

Chamberlin, 1914

########## Published records.

None.

########## Material examined.

**PARÁ**: Canaã dos Carajás (Equatorial Rainforest and “Campos Rupestres”), Iron Ore: Caverna ST 0054, (UFMT) 1 spec, 29.i.2016, BioEspeleo Consultoria Ambiental.

########## Distribution.

This species is typical of northern and western regions, in the states of Amazonas, Pará, and Mato Grosso. Only one occurrence in a cave (iron ore from Pará) was recorded, suggesting that the species is accidental.

########## Habitat.

Unknown.

########## Conservation.

The species’ wide distribution implies that it is not under threat, but the caves of Canaã dos Carajás are part of mining projects.

######### Otostigmus (Parotostigmus) muticus

Taxon classificationAnimaliaScolopendromorphaScolopendridae

Karsch, 1888

########## Published records.

None.

########## Material examined.

**MINAS GERAIS**: Itacarambi (Cerrado-Caatinga transition), Limestone: Lapa da Onça, (UFSCAR) 3 spec, 06.vi.2014, Gallão, JE., von Schimonsky, DM., Monte, BGO.; Lapa do Mogno, (UFSCAR) 1 spec, 12.iv.2015, Gallão, JE., Monte, BGO.

########## Distribution.

*Otostigmus muticus* was recorded in the northern regions of Amazonas and Pará (Schileyko 2002), the extreme west of northeast Maranhão, as well as in the western and central regions of Mato Grosso and Goiás (Chagas-Jr 2012). Specimens were collected from limestone caves in a transitional xeric area of Cerrado and Caatinga (northern Minas Gerais) as well as in southeastern Bahia. The species is likely troglophilic.

########## Habitat.

Unconsolidated substrate, under rocks.

########## Conservation.

This species is not considered threatened due to its wide distribution and the fact that Lapa do Mogno cave, a major habitat, is legally protected as part of the Peruaçu Caves National Park (PCNP) in Minas Gerais.

######### Otostigmus (Parotostigmus) tibialis

Taxon classificationAnimaliaScolopendromorphaScolopendridae

Brölemann, 1902

########## Published records.

None.

########## Material examined.

**SÃO PAULO**: Iporanga (Atlantic Rainforest), Limestone: Gruta da Água Suja, (UFSCAR) 4 spec, 13–20.iv.2009, Pellegatti-Franco, F.; Caverna Guaxica, (UFSCAR) 1 spec, 03.iii.2014, Bichuette, ME., Gallão, JE.; **SANTA CATARINA**: Florianópolis (“Restinga” – Atlantic Forest and coastal vegetation), Granite: Abrigo Saco dos Limões, (UFSCAR) 1 spec, 01.v.2016, Bichuette, ME., Gallão, JE., Lee, D., Xavier, P.; Caverna da Água Corrente, (UFSCAR) 1 spec, 01.v.2016, Bichuette, ME., Gallão, JE., Lee, D., Xavier, P.

########## Distribution.

This species is widely distributed in Brazil, primarily ranging from the central regions and extending southward to Santa Catarina. Also well represented in southeastern and southern Brazil (Chagas-Jr 2012), *O. tibialis* was recorded in limestone and granitic caves of São Paulo and Santa Catarina, respectively. The species is probably troglophilic.

########## Habitat.

Cave (unconsolidated substrate, under rocks).

########## Conservation.

Though widely distributed, the population inhabiting caves of Santa Catarina may be under local threat from urban expansion because there is no relevant legal protection. The species’ cave habitats in São Paulo are, however, located inside state parks.

######### Otostigmus (Parotostigmus) tidius

Taxon classificationAnimaliaScolopendromorphaScolopendridae

Chamberlin, 1914

########## Published records.

None.

########## Material examined.

**MINAS GERAIS**: Diamantina (“Campos rupestres” highland heterogeneous vegetation on rocks), Quartzite: Lapa dos Pombos, (UFSCAR) 1 spec, 06.ix.2013, Fonseca-Ferreira, R.

########## Distribution.

*Otostigmus tidius* is distributed in the central and northern Brazilian states of Tocantins, Mato Grosso, and Goiás, as well as in the Federal District (Brasília) (Chagas-Jr 2012). We provide a new record for the species in Minas Gerais. Accidental in caves.

########## Habitat.

Cave (unconsolidated substrate – sand).

########## Conservation.

*Otostigmus tidius* occurs in a mined cave and is under local threat.

######### Otostigmus (Parotostigmus) spp.

Taxon classificationAnimaliaScolopendromorphaScolopendridae

########## Material examined.

**PARÁ**: Canaã dos Carajás (Equatorial Rainforest and “Campos Rupestres”), Iron Ore: Gruta NV 06, (MNRJ) 1 spec, 22–28.ii.2005, Andrade, R., Arnone, IS.; Gruta S11C 0153, (UFMT) 1 spec, 01.viii.2015, BioEspeleo Consultoria Ambiental; Caverna ST 0003, (UFMT) 1 spec, 21.i.2016, BioEspeleo Consultoria Ambiental; Altamira (Equatorial Rainforest), Sandstone: Caverna Pedra da Cachoeira, (UFSCAR) 1 spec, 03.iv.2011, Bichuette, ME., Gallão, JE., Pedroso, DR., von Schimonsky, DM.; **GOIÁS**: São Domingos (Cerrado), Limestone: Lapa São Bernardo I, (UFSCAR) 1 spec,19.v.2015, Gallão, JE., Paula, CCP.; **MATO GROSSO**: Nobres (Cerrado), Limestone: Toca do Sorvete, (UFSCAR) 1 spec, 06.v.2015, Bichuette, ME., Chagas-Jr, A., Nunes, GA.; **MATO GROSSO DO SUL**: Bodoquena (Cerrado), Limestone: Gruta Dente de Cão , (UFMT) 1 spec, 29.iv.2006; **BAHIA**: Central (Caatinga), Limestone: Toca do Pinguim, (UFSCAR) 1 spec, 19.viii.2015; Gruta da Catedral, (UFSCAR) 2 spec, 19.viii.2015; Andaraí (“Campos rupestres” highland heterogeneous vegetation on rocks), Sandstone: Gruna Rio dos Pombos, (UFSCAR) 1 spec, 10.xii.2015; Catolés (“Campos rupestres” highland heterogeneous vegetation on rocks), Sandstone: Gruna Jambreiro, (UFSCAR) 1 spec, 12.iv.2015, Bichuette, ME.; **MINAS GERAIS**: Itacarambi (Cerrado-Caatinga transition), Limestone: Lapa do Cipó, (UFSCAR) 1 spec, 05.vi.2014, Gallão, JE.; **SANTA CATARINA**: Florianópolis (“Restinga” – Atlantic Forest and coastal vegetation), Granite: Abrigo Saco dos Limões, (UFSCAR) 1 spec, 01.v.2016, Bichuette, ME., Gallão, JE., Lee, D., Xavier, P.

########## Taxonomic notes.

Some specimens lack the ultimate pair of legs, a trait used for species-level identification. Other specimens are different from all described species and are likely to be novel, requiring further examination.

########## Distribution.

Most specimens recorded herein are from limestone caves of Goiás, Mato Grosso, Mato Grosso do Sul, Bahia, and Minas Gerais states. However, some occurrences were reported for iron ore, sandstone, and granitic caves of Pará, Bahia, and Santa Catarina, respectively. Several new occurrences (e.g., at Chapada Diamantina, Bahia) were noted in this study, important for understanding the distribution of cave centipedes.

########## Habitat.

Cave (unconsolidated substrate).

########## Conservation.

Iron ore and granitic caves from Pará and Santa Catarina are under threat from large iron-mining projects and urban expansion, respectively.

####### Genus *Rhysida* Wood, 1862

######## Rhysida brasiliensis

Taxon classificationAnimaliaScolopendromorphaScolopendridae

Kraepelin, 1903

######### Published records.

None.

######### Material examined.

**PARÁ**: Altamira (Equatorial Rainforest), Sandstone: Caverna Pedra da Cachoeira, (UFSCAR) 1 spec,15.xii.2010, Gallão, JE., and (UFSCAR) 1 spec, 03.iv.2011, Bichuette, ME., Gallão, JE., Pedroso, DR., Schimonsky, DM.

######### Distribution.

Among the five Brazilian species of the genus, *R. brasiliensis* is known from southeastern Brazil (Bücherl 1942). New specimens from this study were collected in one sandstone cave of Pará, expanding the species’ record. Accidental in caves.

######### Habitat.

Cave (unconsolidated substrate – sand).

######### Conservation.

The Pedra da Cachoeira cave is located within the boundaries of a huge planned hydroelectrical project (Belo Monte) (M.E. Bichuette, per. obs.). Thus, the species is likely to be threatened on a local scale.

##### Family Cryptopidae Kohlrausch, 1881

###### Subfamily Cryptopinae Kohlrausch, 1881

####### Genus *Cryptops* Leach, 1815

######## Subgenus Cryptops Leach, 1815

######### Cryptops (Cryptops) spelaeoraptor

Taxon classificationAnimaliaScolopendromorphaCryptopidae

Ázara & Ferreira, 2014

########## Published records.

(Ázara and Ferreira 2014a).

########## Material examined.

None.

########## Distribution.

*Cryptops spelaeoraptor* is exclusive to Brazilian caves and only known from the type locality, found in Toca do Gonçalo Cave of Campo Formoso, Bahia. (Ázara and Ferreira 2014a).

########## Habitat.

Cave (under rock, humid substrate).

########## Conservation.

This species is classified as Vulnerable (VU) in the Red List of Brazilian Threatened Fauna (MMA 2016). Toca do Gonçalo Cave has no legal protection from Brazilian environmental agencies. Because it is a rare locale containing water in a semi-arid region, the cave is currently under severe threat from the efforts of local farmers to uncover water resources. Protective measures are urgently required, including monitoring of cave populations and the creation of a conservation unit.

######## Subgenus Trigonocryptops Verhoeff, 1906

######### Cryptops (Trigonocryptops) galatheae

Taxon classificationAnimaliaScolopendromorphaCryptopidae

Meinert, 1886

########## Published records.

None.

########## Material examined.

**SÃO PAULO**: Ribeirão Grande (Atlantic Rainforest), Limestone: Caverna Felício, (UFSCAR) 1 spec, 15.v.2015, Bolfarini, MP.; **SANTA CATARINA**: Florianópolis (“Restinga” – Atlantic Forest and coastal vegetation), Granite: Gruta Pedras Grandes, (UFSCAR) 3 spec, 01.v.2016, Bichuette, ME., Gallão, JE., Lee, D., Xavier, P.

########## Distribution.

This species is widely distributed throughout southern South America. In Brazil, *C. galatheae* is present in all southern states and in São Paulo (Bücherl 1940, 1942). The new records corroborate this distribution and suggest a preference for temperate regions. The species was recorded in one limestone cave and one granitic cave of São Paulo and Santa Catarina, respectively. It is a candidate troglophilic species, but more data are necessary to confirm this categorization.

########## Habitat.

Cave (unconsolidated substrate).

######### Cryptops (Trigonocryptops) iheringi

Taxon classificationAnimaliaScolopendromorphaCryptopidae

Brölemann, 1902

########## Published records.

None.

########## Material examined.

**SÃO PAULO**: Iporanga (Atlantic Rainforest), Limestone: Bairro da Serra, Gruta Ressurgência das Areias de Água Quente, (UFSCAR) 1 spec, vii.1979, Nelson.

########## Distribution.

This species is very common in southeastern and southern Brazil. *Cryptops iheringi* are present in the cities of São Paulo and Curitiba, the former in the downtown area and the latter in home gardens (under or in plant pots) or in landfills (Chagas-Jr et al. 2014). Here we present a record from a limestone cave in São Paulo.

########## Habitat.

Cave (unconsolidated substrate – humid substrate).

######### Cryptops (Trigonocryptops) iporangensis

Taxon classificationAnimaliaScolopendromorphaCryptopidae

Ázara & Ferreira, 2013

########## Published records.

(Ázara and Ferreira 2013).

########## Material examined.

**SÃO PAULO**: Iporanga (Atlantic Rainforest), Limestone: Gruta Monjolinho, (UFSCAR) 1 spec, 09.x.1995, Trajano, E.; and (UFSCAR) 1 spec, 09.v.2005, Trajano, E.; Caverna Alambari de Baixo, (UFSCAR) 1 spec, collected in 02.x.2012, Bichuette, ME.; Caverna Santana, (UFSCAR) 1 spec, 01.i.2012, Bichuette, ME.

########## Distribution.

*Cryptops iporangensis* was described based on a single specimen collected in Ressurgência das Areias de Água Quente Cave, Iporanga, São Paulo. Additional records extend its distribution to three more caves in the Alto Ribeira karst area, suggesting that the species is not endemic to a single cave system or rare, as previously proposed (Ázara and Ferreira 2013). Troglobitic species.

########## Habitat.

Cave (unconsolidated substrate – humid clay, under rocks).

########## Conservation.

New data reinforce the necessity of collections in other caves before establishing cave category (troglobitic, troglophilic, or trogloxene), as these classifications can affect subsequent decisions related to species distribution. For example, *C. iporangensis* was classified as Endangered (EN) (MMA 2016) in the Red List of Brazilian Threatened Fauna because available data suggested that the species was restricted to the Areias cave system. A reevaluation of threat category based on our new data is necessary. Two of the three caves with new *C. iporangensis* records are in limestone outcrops that are not part of the Areias cave system. These outcrops are isolated by non-soluble rocks that limit dispersal of terrestrial cave fauna (Trajano et al. 2016). Furthermore, the species’ cave category should be reviewed given that troglomorphism is not definite.

######### Cryptops (Trigonocryptops) hephaestus

Taxon classificationAnimaliaScolopendromorphaCryptopidae

Ázara & Ferreira, 2013

########## Published records.

(Ázara and Ferreira 2013).

########## Material examined.

Itabirito (“Canga”– heterogeneous flora), Iron Ore: Gruta VL 11, Gruta VL 14, all in Mina Várzea do Lopes, (MNRJ) 1 spec, 18–25.iv.2007, Andrade, R.; Gruta VL 13, Mina Várzea do Lopes, (MNRJ) 2 spec, 18–25.iv.2007, Andrade, R.; Gruta VL 31, Gruta VL 32, all in Mina Várzea do Lopes, (MNRJ) 1 spec, 3–20.xi.2007, Andrade, R.

########## Distribution.

*Cryptops hephaestus* is known from three iron ore caves of the “Quadrilátero Ferrífero” (Iron Quadrangle) in Minas Gerais, one in Mariana municipality and the other two in Itabirito municipality. The latter two caves are close to each other, whereas the former is at least 50 km away. Here, we identified six more specimens from five caves of the Itabirito region. These new records and distributional data (see below) strongly suggest that *C. hephaestus* inhabits areas outside the caves or are present in other caves near the two municipalities. Indeed, the species has no marked troglomorphic traits and should be considered troglophilic.

########## Habitat.

Cave (unconsolidated habitat).

########## Conservation.

*Cryptops hephaestus* occur in caves that are within iron mining areas, severely threatening the species. Urgent conservation action (e.g., monitoring cave fauna) is therefore necessary. The species has not been included in the latest Red List of Brazilian Threatened Fauna (MMA 2016).

######### Cryptops spp.

Taxon classificationAnimaliaScolopendromorphaCryptopidae

########## Material examined.

**PARÁ**: Altamira (Equatorial Rainforest), Sandstone: Caverna Sugiro-Roncador, (UFSCAR) 2 spec, 02.iv.2011, Bichuette, ME., Gallão, JE., Pedroso, DR., von Schimonsky, DM.; Caverna Pedra da Cachoeira, (UFSCAR) 1 spec, 03.iv.2011, Bichuette, ME., Gallão, JE., Pedroso, DR., von Schimonsky, DM.; Canaã dos Carajás (Equatorial Rainforest and “Campos Rupestres”), Iron Ore: Gruta S11C 0060, (UFMT) 1 spec, 05.ix.2015, BioEspeleo Consultoria Ambiental; Gruta S11C 0139, (UFMT) 1 spec, 11.viii.2015, BioEspeleo Consultoria Ambiental; **MATO GROSSO**: Nobres (Cerrado), Limestone: Toca da Serra Rica, (UFSCAR) 1 spec, 04.v.2015, Chagas-Jr, A., Bichuette, ME.; **BAHIA**: Andaraí (“Campos rupestres” highland heterogeneous vegetation on rocks), Sandstone: Gruna Parede Vermelha, (UFSCAR) 1 spec, 29.x.2010, Bichuette, ME., Rantin, B., Gallão, JE.; Gruna do Cantinho, (UFSCAR) 1 spec, 01.iv.2013, Bichuette, ME., von Schimonsky, DM., Gallão, JE.; Gruna Esbirro de Quina, (UFSCAR) 1 spec, 20.x.2014, Gallão, JE., von Schimonsky, DM.; São Desidério (Cerrado-Caatinga transition), Limestone: Caverna Baixa Fria, (UFSCAR) 1 spec, 02.xi.2011, Bichuette, ME.; Carinhanha (Caatinga), Limestone: Gruna da Altina, (UFSCAR) 1 spec, 27.xi.2015, Gallão, JE., Bichuette, ME.; Gruna Valdecir, (UFSCAR) 4 spec, 31.v.2012, Bichuette, ME., Gallão, JE.; Gruna Água Fina, (UFSCAR) 1 spec, 29.v.2012, Bichuette, ME., Gallão, JE.; Paripiranga (Caatinga), Limestone: Caverna do Urutau, (UFSCAR) 1 spec, ix.2013; Abismo do Lixo, (UFSCAR) 1 spec, ix.2013; Caverna da Presa II, (UFSCAR) 2 spec, ix.2013; Abismo dos Morcegos, (UFSCAR) 1 spec, ix.2013, Rocha, KG.; Caverna da Presa II, (UFSCAR) 1 spec, ix.2013;xi.2014, Gallão, JE., Bolfarini, MP., Rosendo, MJ., Moreira, R.; Abismo do Entupido, (UFSCAR) 1 spec, ix.2013; Abismo do Entupido, (UFSCAR) 1 spec, xi.2014, Gallão, JE., Bolfarini, MP., Rosendo, MJ., Moreira, R.; **MINAS GERAIS**: Presidente Olegário (Cerrado and Semideciduous seasonal forest), Limestone: Lapa da Fazenda São Bernardo, (UFSCAR) 1 spec, 23.1.2014, Zepon, T., Resende, LPA.; Lapa da Fazenda São Bernardo, (UFSCAR) 1 spec, 13.iv.2014, Bichuette, ME., Zepon, T., Resende, LPA., Ribeiro, IA.; Lapa da Fazenda São Bernardo, (UFSCAR) 1 spec, 30.xi.2013, Bichuette, ME., Zepon, T., Resende, LPA., Ribeiro, IA.; Montes Claros (Cerrado), Limestone: Lapa da Botina, (UFSCAR) 1 spec, 20.v.2016, Gallão, JE., Zepon, T., von Schimonsky, DM.; Itacarambi (Cerrado-Caatinga transition), Limestone: Lapa da Onça, (UFSCAR) 1 spec, 06.vi.2014, Gallão, JE., von Schimonsky, DM., Monte, BGO.; Gruta Olhos d’ Água, (UFSCAR) 1 spec, 23.x.2013, Bichuette, ME.; São Roque de Minas (Cerrado), Limestone: Gruta do Zeferino I, (UFSCAR) 1 spec, 07.ix.2009, Bichuette, ME.; Pains (Cerrado and Semideciduous seasonal forest), Limestone: Caverna D19, (UFSCAR) 2 spec, 02.xi.2005, Bichuette, ME.; Caverna C13, (UFSCAR) 1 spec, 03.xi.2005, Bichuette, ME.; Diamantina (“Campos rupestres” highland heterogeneous vegetation on rocks), Quartzite: Caverna Tromba D’ Anta, (UFSCAR) 2 spec, 08.ix.2013, Fonseca-Ferreira, R.; Itabirito (“Canga”– heterogeneous flora), Iron Ore: Gruta VL 02, Gruta VL 08, Gruta VL10, all in Mina Várzea do Lopes, (MNRJ) 3 spec, 18–25.iv.2007, Andrade, R.; Gruta VL 03, Gruta VL 60, all in Mina Várzea do Lopes, (MNRJ) 4 spec, 18–25.iv.2007, Andrade, R.; Gruta VL 36 in Mina Várzea do Lopes, (MNRJ) 1 spec, 3–20.xi.2007, Andrade, R.; Gruta VL 18 Mina Várzea do Lopes, (MNRJ)1 spec, iv.2008, Andrade, R.; **SÃO PAULO**: Iporanga (Atlantic Rainforest), Limestone: Caverna Morro Preto, (UFSCAR) 1 spec, 30.ix.2012, Bichuette, M.E.; Caverna Alambari de Baixo, (UFSCAR) 1 spec, 02.x.2012, Bichuette, M.E.; Gruta Betari de Baixo, (UFSCAR) 1 spec, 22.vii.2010, Bichuette, ME., Rizzato, PP., Fernandes, CS.; Gruta das Aranhas, (UFSCAR) 1 spec, 14.vii.2008, Bichuette, ME.; Gruta da Água Suja, (UFSCAR) 1 spec, 16–20.ix.2010 Pellegatti-Franco, F.; Apiaí/Iporanga (Atlantic Rainforest), Limestone: Gruta Mãozinha, (UFSCAR) 1 spec, 14–16.ix.2009, Pellegatti-Franco, F.; Gruta Espírito Santo, (UFSCAR) 1 spec, 08–13.ix.2009, Pellegatti-Franco, F.

########## Taxonomic notes.

*Cryptops* is a common scolopendromorph genus in Brazil but also has worldwide distribution. The genus occurs in caves from Brazil, Spain, Australia, and Cuba (Ribaut 1915, Zapparoli 1990, Edgecombe 2005, 2006, Matic et al. 1977). Most *Cryptops* species recorded in Brazilian caves are troglobitic. As observed for some *Otostigmus* records, several specimens lacked the ultimate pair of legs or were damaged juveniles, making precise identification difficult. At least seven new troglobitic species are currently under study. They are listed here for their important in clarifying cave centipede distribution.

########## Distribution.

Most specimens are from limestone caves of Mato Grosso, Bahia, Minas Gerais, and São Paulo. The remainder are from iron ore and sandstone caves of Pará (northern Brazil), as well as iron ore caves of Minas Gerais (southeastern Brazil). The caves in both states are affected by major iron mining projects, while or hydroelectrical construction also affects caves in Pará.

##### Family Scolopocryptopidae Pocock, 1896

###### Subfamily Scolopocryptopinae Pocock, 1896

####### Genus *Scolopocryptops* Newport, 1844

######## Scolopocryptops ferrugineus macrodon

Taxon classificationAnimaliaScolopendromorphaScolopocryptopidae

(Kraepelin, 1903)

######### Published records.

None.

######### Material examined.

**PARÁ**: Altamira (Equatorial Rainforest), Sandstone and Shale: Caverna Sugiro-Roncador, (UFSCAR) 2 spec, 02.iv.2011, Bichuette, ME., Gallão, JE., Pedroso, DR., von Schimonsky, DM.; Caverna Leonardo da Vinci, (UFSCAR) 2 spec, xii.2010, Bichuette, ME., Gallão, JE., von Schimonsky, DM.; **SÃO PAULO**: Altinópolis (Cerrado and Semideciduous seasonal forest), Sandstone: Caverna Pratinha, (UFSCAR) 1 spec, 31.x.2016, Gallo, JS., Mello, RV., Ferro, JP.

######### Distribution.

Sympatric with *S. miersii*, *S. ferrugineus macrodon* is widely distributed from northern to southern Brazil. Our records indicated occurrence only in sandstone caves from Pará and São Paulo. An accidental species.

######### Habitat.

Cave (unconsolidated substrate – sand, under rocks).

######## Scolopocryptops melanostoma

Taxon classificationAnimaliaScolopendromorphaScolopocryptopidae

Newport, 1845

######### Published records.

None.

######### Material examined.

**PARÁ**: Altamira (Equatorial Rainforest), Shale: Caverna Leonardo da Vinci, (UFSCAR) 1 spec, xii.2011, Bichuette, ME., Gallão, JE., von Schimonsky, DM.

######### Distribution.

The most widespread scolopocryptopid, *S. melanostoma* is a Gondwanan species occurring in the Neotropical, Indo-Malay, and Pacific Island regions (Chagas-Jr 2010). In Brazil, records place the species primarily in the southeast, but it also occurs in Amapá and Pará. The species was sighted in the Leonardo da Vinci shale cave from the latter state. Accidental in caves.

######### Habitat.

Cave (under rocks).

######### Conservation.

The shale cave is located within the boundaries of the Belo Monte hydroelectrical dam and is under threat.

######## Scolopocryptops miersii

Taxon classificationAnimaliaScolopendromorphaScolopocryptopidae

Newport, 1845

######### Published records.

None.

######### Material examined.

**PARÁ**: Parauapebas (Equatorial Rainforest and “Campos Rupestres”), Iron Ore: Gruta N5E 06, Gruta N5E 10, (MNRJ) 2 spec, 22.iii–03.iv.2005, Andrade, R., Arnone, IS.; Gruta N4WS 11 , (MZUSP) 2 spec, 20.x–01.xi.2006, Andrade, R.; Gruta N4E 14, Gruta N4WS 13, Gruta N4E 21, (MZUSP) 3 spec, 20.x–01.xi.2006, Andrade, R.; Gruta N4E 26, (MZUSP) 2 spec, Gruta N4E 33, (MZUSP) 1 spec, 08–12.ii.2007, Andrade, R.; Gruta N1 170 Pereira, (MNRJ) 2 spec, 28.iv–03.x.2007, Andrade, R.; Gruta N1 64, Gruta N1 22 Fael, Gruta N1 02, and Gruta N1 103, (MNRJ) 4 spec, 28.ix–03.x.2007, Andrade, R.; Gruta N1 20 and Gruta N1 75, (MNRJ) 4 spec, 28.ix–03.x.2007, Andrade, R.; Gruta GE 1578 CL Tarzan, (MNRJ) 1 spec, 17–24.x.2008, Andrade, R.; Altamira (Equatorial Rainforest), Sandstone: Caverna Pedra da Cachoeira, (UFSCAR) 2 spec, xii.2010, Bichuette, ME., Gallão, JE., von Schimonsky, DM.; Caverna Pedra da Cachoeira, (UFSCAR) 2 spec, 03.iv.2011, Bichuette, ME., Gallão, JE., Pedroso, DR., von Schimonsky, DM.; Caverna Sugiro-Roncador, (UFSCAR) 1 spec, 02.iv.2011, Bichuette, ME., Gallão, JE., Pedroso, DR., von Schimonsky, DM.; Canaã dos Carajás (Equatorial Rainforest and “Campos Rupestres”), Iron Ore: Gruta S11C 0058, (UFMT) 1 spec, 27.viii.2015, BioEspeleo Consultoria Ambiental; Gruta S11C 0153, (UFMT) 1 spec, 01.viii.2015, BioEspeleo Consultoria Ambiental; Caverna ST 0041, (UFMT) 1 spec, 23.i.2016, BioEspeleo Consultoria Ambiental; **GOIÁS**: Mambaí (Cerrado), Limestone: Gruta da Tarimba, (UFSCAR) 1 spec, 29.iv.2013, Bichuette, ME., Gallão, JE., von Schimonsky, DM., Rizzato, PP., Borghezan, R.; **MATO GROSSO**: Nobres (Cerrado), Limestone: Toca Ronco do Bugio, (UFSCAR) 1 spec, 22.ix.2015, Chagas-Jr, A., Bichuette, ME.; **BAHIA**: Carinhanha (Caatinga), Limestone: Gruna das Três Cobras, (UFSCAR) 1 spec, 30.v.2012, Bichuette, ME., São Desidério, Caverna Baixa Fria, (UFSCAR) 1 spec, 02.ix.2011, Bichuette, ME.; **MINAS GERAIS**: Montes Claros (Cerrado), Limestone: Fenda do Anfiteatro I, (UFSCAR) 1 spec, 22.v.2016, Gallão, JE., Zepon, T., von Schimonsky, DM.; Lapa do Beija Flor, (UFSCAR) 2 spec, 21.v.2016, Gallão, JE., Zepon, T., von Schimonsky, DM.; **SÃO PAULO**: Iporanga (Atlantic Rainforest), Limestone: Gruta do Monjolinho, (UFSCAR) 1 spec, 09.x.2005, Trajano, E.

######### Distribution.

The most widely distributed *Scolopocryptops* in Brazil, *S. miersii* is found in iron ore and sandstone caves of Pará. More commonly, it occurs in limestone and sandstone caves of the central, western, northeast, and southeast regions.

######## Scolopocryptops troglocaudatus

Taxon classificationAnimaliaScolopendromorphaScolopocryptopidae

Chagas-Jr & Bichuette, 2015

######### Published records.

(Chagas-Jr and Bichuette 2015).

######### Material examined.

**BAHIA**: Andaraí (“Campos rupestres” highland heterogeneous vegetation on rocks), Sandstone: Gruna Parede Vermelha, (UFSCAR) 1 spec, 11.ii.2012, Bichuette, ME., Gallão, JE., Giupponi, A.; Gruna Lava Pé, (UFSCAR) 3 spec, 10.iv.2014, (UFSCAR) 2 spec,10.iii.2013, and (UFSCAR) 1 spec, vii.2016, Gallão, JE.; (UFSCAR) 2 spec, 10.iii.2012, Bichuette, ME., Gallão, JE., Giupponi, A.

######### Remarks.

*Scolopocryptops troglocaudatus* is a troglobitic species, morphologically close to *S. miersii* and *S. ferrugineus macrodon*, but is distinguished by troglomorphic features, including depigmentation, long appendages (locomotory and ultimate legs), and thin cuticle (Chagas-Jr and Bichuette 2015).

######### Distribution.

This troglobitic species is likely endemic to Bahia, being only known from siliciclastic (sandstone) caves of the Igatu region (Chapada Diamantina). Its distributional area there is approximately 10 km^2^ (Chagas-Jr and Bichuette 2015).

######### Habitat.

Cave (unconsolidated substrate – sand).

######### Conservation.

A second troglobitic centipede species occurs in the same region (unpublished data, Chagas-Jr), corroborating the hypothesis of an area high in cave-invertebrate diversity (Gallão and Bichuette 2015). Within this region, at least 20 endemic troglobitic species are distributed in a 25 km^2^ area. Although the region is within the Chapada Diamantina National Park (CDNP), small-scale illegal mining persists in the Igatu region and serves as the main threat to cave fauna.

##### Subfamily Newportiinae Pocock, 1896

###### Genus *Newportia* Gervais, 1847

####### Subgenus Newportia Gervais, 1847

######## Newportia (Newportia) ernstiernsti

Taxon classificationAnimaliaScolopendromorphaScolopocryptopidae

Pocock, 1891

######### Published records.

None.

######### Material examined.

**PARÁ**: Parauapebas (Equatorial Rainforest and “Campos Rupestres”), Iron Ore: Gruta N5E 03, (MNRJ) 1 spec, 22.iii–03.iv.2005, Andrade, R., Arnone, IS.; Gruta N4E 02, (MZUSP) 1 spec, Gruta N4E 09, (MZUSP) 1 spec, 20.x–01.xi.2006, Andrade, R.; Gruta N4E 61, (MZUSP) 1 spec, 08–12.ii.2007, Andrade, R.; Gruta S11B 13, (MNRJ) 1 spec, 23.viii–02.ix.2007, Andrade, R.; Gruta N1 15 Mangangá Flona Carajás, (MNRJ) 1 spec, 28.ix–03.x.07, Andrade, R.; Altamira (Equatorial Rainforest), Shale: Caverna Leonardo da Vinci, (UFSCAR) 1 spec, xii.2010, Bichuette, ME., Gallão, JE., von Schimonsky, DM.

######### Distribution.

This is a well-known *Newportia* species occurring on several Antilles islands and in northern South America. In Brazil specifically, *N. ernsti ernsti* has been observed in Amazonas, Pará, and Mato Grosso (Schileyko and Minelli 1999). Cave records follow the species’ distribution pattern, with occurrence in iron ore and sandstone caves of Pará.

######## Newportia (Newportia) ernstifossulata

Taxon classificationAnimaliaScolopendromorphaScolopocryptopidae

Bücherl, 1942

######### Published records.

None.

######### Material examined.

**PARÁ**: Canaã dos Carajás (Equatorial Rainforest and “Campos Rupestres”), Iron Ore: Gruta S11C 0002, (UFMT) 1 spec, 04.viii.2015, BioEspeleo Consultoria Ambiental; Gruta S11C 0135, (UFMT) 1 spec, 05.viii.2015, BioEspeleo Consultoria Ambiental; Caverna ST 0034, (UFMT) 1 spec, 04.ii.2016, BioEspeleo Consultoria Ambiental.

######### Distribution.

*Newportia ernsti fossulata* is known from the northern Brazilian state of Pará (Bücherl 1942, Schileyko and Minelli 1999) and the western state of Mato Grosso (Schileyko and Minelli 1999). Occurrence records are from three iron ore caves that are part of major mining projects in Pará. Accidental in caves.

Newportia (Newportia) lasia Chamberlin, 1921

######### Published records.

None.

######### Material examined.

**PARÁ**: Parauapebas (Equatorial Rainforest and “Campos Rupestres”), Iron Ore: Gruta N4E 33, (MZUSP) 1 spec, 08–12.ii.2007, Andrade, R.; Gruta N5E 08, (MNRJ) 1 spec, 22.iii–03.iv.2005, Andrade, R., Arnone, IS.

######### Distribution data.

This species is known from Guyana, northern Brazil (Amazonas region) and Paraguay (Schileyko and Minelli 1999). Both iron ore caves in which *N. lasia* occurred were within a large iron mining project of Pará. Accidental in caves.

######## Newportia (Newportia) phoreta

Taxon classificationAnimaliaScolopendromorphaScolopocryptopidae

Chamberlin, 1950

######### Published records.

None.

######### Material examined.

**PARÁ**: Parauapebas (Equatorial Rainforest and “Campos Rupestres”), Iron Ore: Gruta N4E 005, (UFMT) 1 spec, 13.ii.2014, BioEspeleo Consultoria Ambiental; Gruta N4E 26, (MZUSP) 1 spec, 08–12.ii.2007, Andrade, R.

######### Distribution.

*Newportia phoreta* is known from observations in Venezuela (Schileyko and Minelli 1999) and Colombia (Chagas-Jr et al. 2014), with the current study being the first Brazilian record. The species was found in only two iron ore caves from Pará, both part of a large huge iron mining project.

######### Conservation.

The new record and restricted distribution in Brazil put the species under a high level of local threat within the Carajás region.

######## Newportia (Newportia) potiguar

Taxon classificationAnimaliaScolopendromorphaScolopocryptopidae

Ázara & Ferreira, 2014

######### Published records.

(Ázara and Ferreira 2014b).

######### Material examined.

None.

######### Taxonomic notes.

*Newportia potiguar* is a recently described species, with two small specimens characterized by marked troglomorphism: elongation of the ultimate legs (half of the body length) and antennae, cuticle sclerotization, as well as reduced pigmentation (Ázara and Ferreira 2014b). (Even without the latter characteristic, the former two traits are sufficient for troglomorphic characterization) Indeed, juveniles of the troglobitic *S. troglocaudatus* are completely pale, even in the appendages (Chagas-Jr and Bichuette 2015). Therefore, the character of reduced pigmentation should be investigated in other juvenile centipedes to properly interpret its application as a troglomorphic trait. *Newportia potiguar* resembles *N. brevipes* Pocock, 1891, but is closely related to *N. stolli* (Pocock, 1896) based on morphology of the ultimate and locomotory legs. The former exhibits four spinous processes on the prefemur and two on the femur; the latter has ventral, lateral, and tarsal spurs (Ázara and Ferreira 2014b). Distinct from *N. stolli*, *N. potiguar* has posterior transverse sutures on tergite 1 and paramedian sutures on tergite 2. Although considered a troglobitic species, no collections were conducted outside the cave to confirm this categorization.

######### Distribution.

This species is known only from two limestone caves of Rio Grande do Norte, a semi-arid state in northeastern Brazil (Ázara and Ferreira 2014b).

######### Habitat.

Cave (under rocks – humid substrate).

######### Conservation.

The caves are not under legal protection. Primary threats are exploration for petroleum and illegal limestone extraction. *Newportia potiguar* was not evaluated in the last Red List of Brazilian Threatened Fauna (MMA 2016).

######## Newportia (Newportia) spelaea

Taxon classificationAnimaliaScolopendromorphaScolopocryptopidae

Ázara & Ferreira, 2014

######### Published records.

(Ázara and Ferreira 2014b).

######### Material examined.

None.

######### Taxonomic notes.

The small body size suggests that the specimen was a juvenile. Thus, more collections are necessary to confirm diagnostic characters and cave category.

######### Distribution.

*Newportia spelaea* is known only from a single specimen from Toca do Gonçalo, a limestone cave in the semi-arid, northeastern Brazilian state of Bahia. The species is classified as troglobitic, but few collections were conducted outside the cave to confirm this categorization.

######### Habitat.

Cave (humid substrate).

######### Conservation.

Toca do Gonçalo has no legal protection from Brazilian environmental agencies and is under severe threat by local people for the water resources within. *Newportia spelaea* was not evaluated in the Red List of Brazilian Threatened Fauna (MMA 2016).

######## Newportia (Newportia) spp.

Taxon classificationAnimaliaScolopendromorphaScolopocryptopidae

######### Material examined.

**PARÁ**: Parauapebas (Equatorial Rainforest and “Campos Rupestres”), Iron Ore: Gruta N4E 22, (MZUSP) 1 spec, 20.x–01.xi.2006, Andrade, R.; Gruta N4E 32, (MZUSP) 1 spec, 22.iii–03.iv.2006, Andrade, R., Arnone, IS.; Gruta GEM 154 CL Tarzan, (MNRJ) 1 spec, 17–24.x.2008, Andrade, R.; Gruta S11A 12, ((MNRJ) 1 spec, 23.viii–02.ix.2007, Andrade, R.; Gruta N4E 30, (MZUSP) 1 spec, 08 12.ii.2007, Andrade, R.; Gruta N4E 11, (MZUSP) 2 spec, 20.x–01.xi.2006, Andrade, R.; Canaã dos Carajás (Equatorial Rainforest and “Campos Rupestres”), Iron Ore: Caverna ST 0030 (4), (UFMT) 1 spec, 04.ii.2016, BioEspeleo Consultoria Ambiental; Altamira (Equatorial Rainforest), Sandstone: Caverna Pedra da Cachoeira, (UFSCAR) 3 spec, 03.iv.2011, Bichuette, ME.; Abrigo do Abutre, (UFSCAR) 1 spec, 11.iv.2009, Bichuette, ME.; Caverna Pedra da Cachoeira, (UFSCAR) 1 spec, 03.iv.2011, Bichuette, ME., Gallão, JE., Pedroso, DR., von Schimonsky, DM.; Caverna Sugiro-Roncador, (UFSCAR) 1 spec, 02.iv.2011, Bichuette, ME., Gallão, JE., Pedroso, DR., Schimonsky, DM.; **MATO GROSSO**: Nobres (Cerrado), Limestone: Duto do Quebó, (UFSCAR) 1 spec, 23.ix.2015, Chagas-Jr, A., Bichuette, ME.; **CEARÁ**: Crato (Caatinga), Limestone: Pedra Fedorena, (UFSCAR) 1 spec, 15.vii.2002, Trajano, E., Bichuette, ME., Souza, L.; **BAHIA**: Paripiranga (Cattinga), Limestone: Caverna do Alto do Morro da Candeia, (UFSCAR) 1 spec, xi.2014, Gallão, JE., Bolfarini, MP., Rosendo, MJ., Moreira, R.

######### Taxonomic notes.

These specimens were damaged or lacked the ultimate pair of legs, precluding proper identification. They likely belong to more than one morphospecies. Mitochondrial sequence data might be a useful alternative for identifying *Newportia* species because it can accurately classify damaged specimens (Edgecombe et al. 2015). These damaged specimens are listed for clarification of cave-centipede distribution.

######### Distribution.

The genus *Newportia* is widely distributed in the Neotropics, from central Mexico, through the Greater/Lesser Antilles, and occupying almost all of South America down to Uruguay. Records are from iron ore caves of Pará, sandstone caves of Mato Grosso, as well as limestone caves of Ceará and Bahia.

####### Subgenus Tidops Chamberlin, 1915

######## Newportia (Tidops) balzanii

Taxon classificationAnimaliaScolopendromorphaScolopocryptopidae

Silvestri, 1895

######### Published records.

None.

######### Material examined.

**GOIÁS**: São Domingos (Cerrado), Limestone: Lapa São Bernardo I, (UFSCAR) 1 spec, 19.v.2015, Gallão, JE., Paula, CCP; Lapa São Bernardo II, (UFSCAR) 1 spec, 21.v.2015, Bichuette, ME.; Mambaí (Cerrado), Limestone: Gruta da Tarimba, (UFSCAR) 1 spec, 29.iv.2013, Bichuette, ME., Gallão, JE., von Schimonsky, DM., Rizzato, PP., Borghezan, R.; **BAHIA**: Paripiranga (Caatinga), Limestone: Caverna das Moscas, (UFSCAR) 1 spec, in xi.2014, Gallão, JE., Bolfarini, MP., Rosendo, MJ., Moreira, R.; Central (Caatinga):, Limestone Gruta do Tonho, (UFSCAR) 1 spec, 02.ii.2015, Gallão, JE., Bichuette, ME.; **MINAS GERAIS**: Itacarambi (Cerrado-Caatinga transition), Limestone: Gruta Olhos d’Água, (UFSCAR) 1 spec, 23.x.2013, Bichuette, ME.; Lapa do Branco I, (UFSCAR) 1 spec, vi.2014, Gallão, JE., von Schimonsky, DM., Monte, BGO; Lapa do Mogno, (UFSCAR) 1 spec, 12.iv.2015, Gallão, JE., Monte, BGO.; Lapa Mina d’Água, (UFSCAR) 2 spec, 10.iv.2015, Gallão, JE., Monte, BGO.

######### Distribution.

This species is distributed in central, western, and southwestern South America, encompassing Brazil, Bolivia, and Paraguay (Chagas-Jr 2011). Records in Brazil originate from Tocantins, Goiás, and São Paulo e Rio Grande do Sul. The new data presented here place the species in limestone caves of Goiás, Bahia, and Minas Gerais, extending its distribution to two more states.

######## Newportia (Tidops) nisargani

Taxon classificationAnimaliaScolopendromorphaScolopocryptopidae

(Chagas-Jr, 2011)

######### Published records.

None.

######### Material examined.

**PARÁ**: Parauapebas (Equatorial Rainforest and “Campos Rupestres”), Iron Ore: Gruta N4E 0036, (UFMT) 1 spec, 14.ii.2014, BioEspeleo Consultoria Ambiental; Canaã dos Carajás (Equatorial Rainforest and “Campos Rupestres”), Iron Ore: Gruta S11C 0102, (UFMT) 1 spec, 03.xi.2015, BioEspeleo Consultoria Ambiental; Caverna ST 0037, (UFMT) 1 spec, 25.i.2016, BioEspeleo Consultoria Ambiental; **BAHIA**: Carinhanha (Caatinga): Gruna Água Fina, (UFSCAR) 1 spec, 29.v.2012, Bichuette, ME., Gallão, JE.; **MINAS GERAIS**: Montes Claros (Cerrado), Limestone: Lapa do Ninho, (UFSCAR) 1 spec, 23.v.2016 by Gallão, JE., Zepon, T., von Schimonsky, DM.

######### Distribution.

This species is known from the states of Amazonas, Pará, and Bahia. Observed in iron ore caves from Pará, as well as in limestone caves of Bahia and Minas Gerais (the latter rarely), *N. nisargani* distribution has now increased to southeastern Brazil. Despite intensively sampling, only one specimen was recorded from Minas Gerais, in a cave of the Montes Claros municipality.

######## Newportia (Tidops) simus

Taxon classificationAnimaliaScolopendromorphaScolopocryptopidae

Chamberlin, 1950

######### Published records.

None.

######### Material examined.

**PARÁ**: Altamira (Equatorial Rainforest), Sandstone: Abrigo da Gravura, (UFSCAR) 1 spec, 08.vii.2009, Bichuette, M.E.

######### Distribution.

This species is known from Grenada, Lesser Antilles, and Brazil (Santarém, Pará). The current study noted its occurrence in a sandstone cave from Pará (Altamira region).

######### Habitat.

Cave (unconsolidated substrate – sand).

######### Conservation.

This cave was flooded by the Belo Monte reservoir.

######## Newportia (Tidops) spp.

Taxon classificationAnimaliaScolopendromorphaScolopocryptopidae

######### Material examined.

**PARÁ**: Canaã dos Carajás (Equatorial Rainforest and “Campos Rupestres”), Iron Ore: Gruta S11C 0007, (UFMT) 1 spec, 04.viii.2015 by BioEspeleo Consultoria Ambiental; **MINAS GERAIS**: Caeté (“Canga”– heterogeneous flora), Iron Ore: Gruta da Piedade, (UFSCAR) 1 spec, 15.xii.2012, Bichuette, ME., Faria, LE., Gallão, JE., Rocha, A., Fonseca, T..

######### Distribution.

Newportia (Tidops) is a typical newportiine from South America, with some records in the Lesser Antilles. Unidentified specimens in this study were collected from iron ore and limestone caves of Pará and Minas Gerais, respectively. Records are included to clarity cave-centipede distribution.

######## Order Geophilomorpha

Taxon classificationAnimaliaScolopendromorphaScolopocryptopidae

Pocock, 1895

######### Material examined.

**PARÁ**: Parauapebas (Equatorial Rainforest and “Campos Rupestres”), Iron Ore: Gruta N5S S652, (MNRJ) 1 spec, 30.1–30.iv.2012, Andrade, R.; Gruta N5S S642, (MNRJ) 1 spec, 30.1–30.iv.2012, Andrade, R.; Gruta N4E 12, (MZUSP) 1 spec, 20.x–01.xi.2006, Andrade, R.; Gruta N4E 16, (MZUSP) 1 spec, 20.x–01.xi.2006, Andrade, R.; **MATO GROSSO DO SUL**: Bonito (Cerrado), Limestone: Gruta João Arruda, (UFSCAR) 1 spec, 21.vii.1991, Trajano, E.; **BAHIA**: Paripiranga (Caatinga), Limestone: Abismo da Bezerra, (UFSCAR) 1 spec, xi.2014, Gallão, JE.; Ourolandia (Caatinga), Limestone: Toca da Nicinha, (UFSCAR) 1 spec, 17.vi.2015, von Schimonsky, DM.; Carinhanha (Caatinga), Limestone: Gruna Valdecir, (UFSCAR) 1 spec, 31.v.2012, Bichuette, ME., Gallão, JE.; **MINAS GERAIS**: Presidente Olegário (Cerrado and Semideciduous seasonal forest), Limestone: Lapa da Fazenda São Bernardo, (UFSCAR) 2 spec, 10.vi.2014, Zepon, T., Resende, LPA.; (UFSCAR) 2 spec, 30.xi.2013, Bichuette, ME., Zepon, T., Resende, LPA., Ribeiro, IA.; (UFSCAR) 3 spec, 13.iv.2014, Bichuette, ME., Zepon, T., Resende, LPA., Ribeiro, IA.; Lapa do Moacir, (UFSCAR) 1 spec, 17.iv.2014, Zepon, T., Resende, LPA., Damasceno, GF.; Caeté (“Canga”– heterogeneous flora), Iron Ore: Gruta da Piedade, (UFSCAR) 1 spec, 07.vi.2013, Bichuette, ME., Faria, LE., Gallão, JE., Rocha, A., Fonseca, T.; Itabirito (“Canga”– heterogeneous flora), Iron Ore: Gruta VL 15 Mina Várzea do Lopes, (MNRJ) 1 spec, 18–25.iv.07, Andrade, R.; **SÃO PAULO**: Apiaí/Iporanga (Atlatinc Rainforest), Limestone: Caverna Arataca, (UFSCAR) 1 spec, 08–13.ix.2009, Pellegatti-Franco, F.; Gruta Toca dos Meninos, (UFSCAR) 1 spec, 14 16.ix.2009, Pellegatti-Franco, F.; Iporanga (Atlantic Rainforest), Limestone: Caverna do Couto, 1 spec, 13–20.iv.2009 Pellegatti-Franco, F.

######### Distribution.

The distribution of Brazilian geophilomorphs remains poorly understood. A few early and mid-twentieth-century studies on Brazilian centipedes reported geophilomorphs (Brölemann 1909; Chamberlin 1914, Bücherl, 1940, 1942), but more recent studies tended to focus on Amazonian fauna (Calvanese and Brescovit 2017, Foddai et al. 2002, Pereira 2012, 2013, Pereira et al. 1994, 1995, 2000). The first geophilomorph records in Brazilian caves are from limestone caves in Mato Grosso do Sul (Dessen et al. 1980) and São Paulo (Gnaspinni and Trajano 1994, Trajano 1987). Unidentified geophilomorphs examined here expands their distribution to iron ore caves from Pará and Minas Gerais, as well as to limestone caves of Mato Grosso do Sul, Bahia, Minas Gerais, and São Paulo. Records are included to clarify centipede distribution.

######## Family Ballophilidae

Taxon classificationAnimaliaScolopendromorphaScolopocryptopidae

Cook, 1896

######### Material examined.

**PARÁ**: Parauapebas (Equatorial Rainforest and “Campos Rupestres”), Iron Ore: Gruta N4E 22 CL, (MNRJ) 1 spec, 7–12.x.2008, Andrade, R.; Gruta N4E 08, (MZUSP) 1 spec, 20.x–01.xi.2006, Andrade, R.; Gruta N4E 32, (MZUSP) 1 spec, 08–12.ii.2007, Andrade, R.; Canaã dos Carajás (Equatorial Rainforest and “Campos Rupestres”), Iron Ore: Gruta NV 09, (MNRJ) 1 spec, 22–28.ii.2006, Andrade, R., Arnone, IS.; Gruta S11C 0060, (UFMT) 1 spec, 05.ix.2015, BioEspeleo Consultoria Ambiental; Caverna ST 0054, (UFMT) 1 spec, 29.i.2016, BioEspeleo Consultoria Ambiental; **GOIÁS**: São Domingos (Cerrado), Limestone: Lapa São Bernardo II, (UFSCAR) 1 spec, 19.v.2015, Bichuette, ME.; **BAHIA**: Paripiranga (Caatinga), Limestone: Caverna do Alto do Morro da Candeia, (UFSCAR) 3 spec, 25.xi.2014, Gallão, JE., Bolfarini, MP., Rosendo, MJ., Moreira, R.; Furna Sem Nome, (UFSCAR) 1 spec, xi.2014, Gallão, JE., Bolfarini, MP., Rosendo, MJ., Moreira, R.; **MINAS GERAIS**: Itabirito (“Canga”– heterogeneous flora), Iron Ore: Gruta VL 32 Mina Várzea do Lopes, (MNRJ) 1 spec, 3–20.xi.07, Andrade, R.

######### Taxonomic notes.

These specimens were only identified to the family level because some were juveniles, while others were damaged or possibly unknown species that should be examined in greater detail.

######### Distribution.

Brazil (specifically Amazonas and Rio de Janeiro states) has eight Ballophilidae species from the genera *Ityphilus* Cook, 1899 and *Taeniolinum* Pocock, 1894 (Bonato et al. 2016). This study is the first to report ballophilid species in Brazilian caves. Most known Brazilian species in this family are from the Amazonian Forest, except *Ityphilus bonatoi* Pereira, 2013 from the Atlantic Rainforest of Rio de Janeiro. This study presents further records in caves of Pará (iron ore), Minas Gerais (iron ore), as well as Goiás and Bahia (limestone caves).

###### Genus *Ityphilus* Cook, 1899

####### Ityphilus spp.

Taxon classificationAnimaliaScolopendromorphaScolopocryptopidae

######## Material examined.

**PARÁ**: Parauapebas (Equatorial Rainforest and “Campos Rupestres”), Iron Ore: Gruta N4E 0026, (UFMT) 1 spec, 22.i.2015, BioEspeleo Consultoria Ambiental; Canaã dos Carajás (Equatorial Rainforest and “Campos Rupestres”), Iron Ore: Caverna ST 0054, (UFMT) 1 spec, 29.i.2016, BioEspeleo Consultoria Ambiental; **BAHIA**: Paripiranga (Caatinga), Limestone: Caverna do Alto do Morro da Candeia, (UFSCAR) 1 spec, ix.2013, Rocha, KG.

######## Distribution.

*Ityphilus* is the most species-rich and widespread ballophilid genera in the Neotropics (Pereira 2013). Seven species were recorded in Brazil, six from the Amazonian Forest (Amazonas), and one from the Atlantic Rainforest (Rio de Janeiro). Here, *Ityphilus* was observed for the first time in caves iron caves of Pará and a limestone cave of Bahia).

####### Family Geophilidae

Taxon classificationAnimaliaScolopendromorphaScolopocryptopidae

Leach, 1815

######## Material examined.

**PARÁ**: Parauapebas (Equatorial Rainforest and “Campos Rupestres”), Iron Ore: Gruta S11D 64, (MNRJ) 6 spec, 23.viii–02.ix.2007, Andrade, R.; Gruta N5S 18, (MNRJ) 1spec, 22.iii–03.iv.2005, Andrade, R., Arnone, IS.; Gruta N4E 16, (MZUSP) 1spec, 20.x–01.xi.2006, Andrade, R.; Gruta N5S 37 CL, (MNRJ) 1spec, 7–12.x.2008, Andrade, R.; Canaã dos Carajás (Equatorial Rainforest and “Campos Rupestres”), Iron Ore: Caverna ST 0041, (UFMT) 1spec, 23.i.2016, BioEspeleo Consultoria Ambiental; Curionópolis (Equatorial Rainforest and “Campos Rupestres”), Iron Ore: Gruta SL 74 CL, (MNRJ) 1spec, 17–24.x.2008, Andrade, R.; Altamira (Equatorial Rainforest), Sandstone and Shale: Abrigo Assurini, (UFSCAR) 1spec, xii.2010, Bichuette, ME., Gallão, JE., von Schimonsky, DM.; Caverna Leonardo da Vinci, (UFSCAR) 1 spec, 14.iv.2009, Bichuette, ME.; **SERGIPE**: Japaratuba (“Restinga” – Atlantic Forest and coastal vegetation), Limestone: Caverna Casa do Caboclo, (UFSCAR) 1 spec, 19.x.2014, Bichuette, ME.; **MINAS GERAIS**: Presidente Olegário (Cerrado and Semideciduous seasonal forest), Limestone: Lapa da Fazenda São Bernardo, (UFSCAR) 1 spec, 13.iv.2014, Bichuette, ME., Zepon, T., Resende, LPA., Ribeiro, IA.

######## Taxonomic notes.

With nearly 560 species representing 100 genera, Geophilidae is a highly diverse family of Geophilomorpha, distributed worldwide (Bonato 2011). Brazil is known to have nine geophilid species from four genera: *Hyphydrophilus* Pereira, Minelli & Barbieri, 1994 (two species), *Ribautia* Brölemann, 1909 (five species), *Schizonampa* Chamberlin, 1914 (one species), and *Sogona* Chamberlin, 1912 (one species). Specimens in the current study were only identified to the family level because some were juveniles, while others damaged or potentially unknown species that require further detailed investigation.

######## Distribution.

One Brazilian Geophilidae occurrence was recorded in the Alto Ribeira karst area of São Paulo, but the genus was not defined (Trajano and Bichuette 2010). Here, we recorded specimens from iron ore and sandstone caves in Pará, as well as from limestone caves of Sergipe and Minas Gerais, extending the family’s distribution to northeastern (Sergipe) and southeastern Brazil (Minas Gerais).

###### Genus *Hyphydrophilus*, Pereira, Minelli & Barbieri, 1994

####### Hyphydrophilus spp.

Taxon classificationAnimaliaScolopendromorphaScolopocryptopidae

######## Material examined.

**PARÁ**: Parauapebas (Equatorial Rainforest and “Campos Rupestres”), Iron Ore: Gruta N4E 0037, (UFMT) 1 spec, 14.ii.2014, BioEspeleo Consultoria Ambiental; Gruta N4WS 17, (MNRJ) 1 spec, 20.x–01.xi.2006, Andrade, R.; Gruta S11 B13E, (MNRJ) 1 spec, 23.viii–02.ix.2007, Andrade, R.

######## Distribution.

Two valid species of *Hyphydrophilus* species are known so far: *H. adisi* Pereira, Minelli & Barbieri, 1994 and *H. projectus* Pereira, Foddai & Minelli, 2000, both from the Amazonian Forest (Amazonas). We now add a record in iron ore caves from Pará, increasing the genus’ distribution down to the southern Amazonian region.

###### Genus *Ribautia* Brölemann, 1909

####### Ribautia spp.

Taxon classificationAnimaliaScolopendromorphaScolopocryptopidae

######## Material examined.

**BAHIA**: Carinhanha (Caatinga), Limestone: Caverna Bem Bom, (UFSCAR) 1 spec, 06.xii.2012, Bichuette, ME., Gallão, JE.; **MINAS GERAIS**: Januária (Cerrado-Caatinga transition), Limestone: Gruta do Janelão, (UFSCAR) 3 spec, 22.vii.2012, Bichuette, ME., Gallão, JE., Rizzato, PP.; **SÃO PAULO**: Apiaí/Iporanga (Atlantic Rainforest), Limestone: Gruta Mãozinha, (UFSCAR) 1 spec, 26–30.iii.2009, Pellegatti-Franco, F.; Gruta Espírito Santo, (UFSCAR) 1 spec, 08–13.ix.2010, Pellegatti-Franco, F.; Iporanga (Atlantic Rainforest), Limestone: Gruta da Água Suja, (UFSCAR) 1 spec, 16–20.ix.2009, Pellegatti-Franco, F.; Caverna do Couto, (UFSCAR) 1 spec, 16–20.ix.2009, Pellegatti-Franco, F.; Gruta Areias de Cima, (UFSCAR) 2 spec, 25.ix.1989, Trajano, E.; Gruta Areias de Cima, (UFSCAR) 1 spec, 30.iv.1990, Trajano, E.; Gruta Casa de Pedra, (UFSCAR) 1 spec, 29.iv.1990, Trajano, E.; Caverna Ressurgência das Areias de Água Quente, (UFSCAR) 1 spec, 15.vi.1991, Trajano, E.; Caverna Laje Branca, (UFSCAR) 1 spec, 23.iv.1992, Trajano, E.; Caverna Guaxica, (UFSCAR) 2 spec, 03.iii.2014, Bichuette, ME., Gallão, JE.

######## Distribution.

There are five species of *Ribautia* in Brazil, all from the state of Amazonas (Bonato et al. 2016). Our records increased *Ribautia* distribution to northeastern and southeastern Brazil, all in limestone caves from Bahia, Minas Gerais, and São Paulo.

######## Conservation.

No major threats affect the relevant caves. Those in Minas Gerais and São Paulo are within conservation units (Peruaçu Caves National Park and Alto Ribeira Touristic State Park, respectively). However, the Serra do Ramalho region (Carinhanha, Bahia) is among the most important areas for troglobitic fauna in Brazil (ME Bichuette in preparation), but the area remains legally unprotected. Further, due to the potential for ore extraction (e.g., niobium), international mining companies are prospecting the region.

###### Genus *Schizonampa* Chamberlin, 1914

####### Schizonampa

Taxon classificationAnimaliaScolopendromorphaScolopocryptopidae

sp.

######## Material examined.

**PARÁ**: Altamira (Equatorial Rainforest), Shale: Caverna Leonardo da Vinci, (UFSCAR) 1 spec, 17.xii.2010, Gallão, JE.

######## Distribution.

Only one *Schinozampa* record exists for Brazil (*Schinozampa mani* Chamberlin, 1914), in Pará (Chamberlin 1914). Our records confirm the genus’ presence in this state, based on a specimen from a sandstone cave of Altamira, Pará. However, this specimen is not conspecific with *S. mani* and may be a novel species that requires further investigation.

####### Family Oryidae

Taxon classificationAnimaliaScolopendromorphaScolopocryptopidae

Cook, 1896

######## Material examined.

**SÃO PAULO**: Apiaí/Iporanga (Atlantic Rainforest), Limestone: Gruta do Minotauro, (UFSCAR) 1 spec, 14–16.ix.2010, Pellegatti-Franco, F.

######## Distribution.

Four Oryidae species are known from Brazil, all belonging to two genera: *Orphnaeus* Meinert, 1870 and *Notiphilides* Latzel, 1880. Oryid species have also been found to the north (Bücherl 1940, 1942, Calvanese and Brescovit 2017) (Amazonas), northeast (Rio Grande do Norte), and southeast (Minas Gerais and São Paulo states) (Bücherl 1940, 1942).

###### Genus *Orphnaeus* Meinert, 1870

####### Orphnaeus spp.

Taxon classificationAnimaliaScolopendromorphaScolopocryptopidae

######## Material examined.

**PARÁ**: Parauapebas (Equatorial Rainforest and “Campos Rupestres”), Iron Ore: Gruta N4E 0057, (UFMT) 1 spec, 25.vii.2015, BioEspeleo Consultoria Ambiental; **BAHIA**: Andaraí (“Campos rupestres” highland heterogeneous vegetation on rocks), Sandstone: Gruna sem nome, (UFSCAR) 1 spec, 19.x.2014, Gallão, JE., von Schimonsky, DM.; Gruna Lava Pé, (UFSCAR) 1 spec, 23.x.2014, Gallão, JE., von Schimonsky, DM.

######## Distribution.

The two Brazilian species (*O. brasilianus* Humbert & Saussure, 1879) was recorded in Rio Grande do Norte and Mato Grosso, and (*O. porosus* Verhoeff, 1937) was recorded in Minas Gerais and São Paulo (Bücherl 1942, Bonato et al. 2016). This study presents the first occurrence of this genus in Brazilian caves.

####### Family Schendylidae

Taxon classificationAnimaliaScolopendromorphaScolopocryptopidae

Cook, 1896

######## Material examined.

**PARÁ**: Parauapebas (Equatorial Rainforest and “Campos Rupestres”), Iron Ore: Gruta N5S S652, (MNRJ) 1 spec, 30.1–30.iv.2012, Andrade, R.; Gruta N5S N701, (MNRJ) 1 spec, 30.1–30.iv.2012, Andrade, R.; Gruta N4E 13, (MNRJ) 1 spec, 20.x–01.xi.2006, Andrade, R.; Altamira (Equatorial Rainforest), Shale: Caverna Leonardo da Vinci, (UFSCAR) 1 spec, 08.vii.2009, Pedroso, DR.; **GOIÁS**: São Domingos (Cerrado), Limestone: Lapa São Vicente II, (UFSCAR) 1 spec, 06.iv.2016, Bichuette, ME.; Mambaí, Gruta da Tarimba, (UFSCAR) 2 spec, 29.iv.2013, Bichuette, ME., Gallão, JE., von Schimonsky, DM., Rizzato, PP., Borghezan, R.; **BAHIA**: Paripiranga (Caatinga), Limestone: Caverna do Escondido, (UFSCAR) 1 spec, ix.2013, Rocha, KG.; Caverna do Alto do Morro da Candeia, (UFSCAR) 1 spec, ix.2013, Rocha, KG; (UFSCAR) 1 spec, xi.2014, Gallão, JE., Bolfarini, MP., Rosendo, MJ., Moreira, R.; Ourolandia (Caatinga), Limestone: Gruta da Fazenda Caldeirão, (UFSCAR) 1 spec, 17.vi.2015, von Schimonsky, DM.; Itaetê (Caatinga), Limestone: Lapa do Bode, (UFSCAR) 1 spec, 21.x.2014, Gallão, JE.; Carinhanha (Caatinga), Limestone: Gruna Valdecir, (UFSCAR) 1 spec, 31.v.2012, Bichuette, ME., Gallão, JE.; **MINAS GERAIS**: Itacarambi (Cerrado-Caatinga transition), Limestone: Gruta Olhos d’ Água, (UFSCAR) 1 spec, 25.iii.2014, Monte, BGO.; Presidente Olegário (Cerrado and Semideciduous seasonal forest), Limestone: Lapa do Moacir, (UFSCAR) 1 spec, 17.iv.2014, Zepon, T., Resende, LPA.; Lapa da Fazenda São Bernardo, (UFSCAR) 1 spec, 22.vii.2012, Bichuette, ME.; Lapa da Fazenda São Bernardo, (UFSCAR) 1 spec, 13.iv.2014, Bichuette, ME., Zepon, T., Resende, LPA., Ribeiro, IA.; **SÃO PAULO**: Iporanga (Atlantic Rainforest), Limestone: Caverna Alambari de Baixo, (UFSCAR) 1 spec, 02.x.2012, Bichuette, M.E.; Gruta Casa de Pedra, (UFSCAR) 1 spec, 29.iv.1990, Trajano, E.; Caverna Passoca de Cima, (UFSCAR) 1 spec, 03.viii.2013, Bichuette, ME.; **SANTA CATARINA**: Florianópolis (“Restinga” – Atlantic Forest and coastal vegetation), Granite: Gruta da Laje, (UFSCAR) 1 spec, 29–30.ix.2016, Gallão, JE., Xavier, P.

######## Distribution.

Schendylidae is the most diverse geophilomorph family in Brazil, with 22 species distributed across two genera, *Pectiniunguis* Bollman, 1889 (six species) and *Schendylops* Cook, 1899 (16 species). Brazilian Schendylidae are mainly found in the north (Amazonas, Amapá, and Pará) and in the southeast (São Paulo and Rio de Janeiro). Records also exist from the northeastern state of Paraíba and in the southern state of Santa Catarina (Bonato et al. 2016). Our materials expanded the family’s range to Goiás (central Brazil) and Bahia (northeastern Brazil). No schendylid species was previously known from Brazilian caves, but we identified specimens in iron ore, sandstone, and shale caves (Pará), limestone caves (Goiás, Bahia, Minas Gerais, and São Paulo), and a granitic cave (Santa Catarina).

###### Genus *Schendylops* Cook, 1899

####### Schendylops spp.

Taxon classificationAnimaliaScolopendromorphaScolopocryptopidae

######## Material examined.

**PARÁ**: Parauapebas (Equatorial Rainforest and “Campos Rupestres”), Iron Ore: Gruta N5S 37 CL, (MNRJ) 1 spec, 07–12.x.2008, Andrade, R. et al.; Gruta N4E 05, (MZUSP) 2 spec, 20.x–01.xi.2006, Andrade, R.; Gruta S11B 11, (MNRJ) 9 spec, 23.viii–02.ix.2007, Andrade, R.; Gruta S11D 64, (MNRJ) 4 spec, 23.viii–02.ix.2007, Andrade, R.; Gruta S11 B13E, (MNRJ) 1 spec, 23.viii–02.ix.2007, Andrade, R.; Gruta N4WS 17, (MZUSP) 1 spec, 20.x–01.xi.2006, Andrade, R.; Gruta N5S 10 CL, (MNRJ) 2 spec, 7–12.x.2008, Andrade, R.; Gruta N4E 33, (MZUSP) 1 spec, 08–12.ii.2007, Andrade, R.; Canaã dos Carajás (Equatorial Rainforest and “Campos Rupestres”), Iron Ore: Gruta S11C 0203 0214, (UFMT) 3 spec, 31.vii.2015, BioEspeleo Consultoria Ambiental; Altamira (Equatorial Rainforest), Shale: Caverna Leonardo da Vinci, (UFSCAR) 3 spec, xii.2010, Bichuette, ME., Gallão, JE., von Schimonsky, DM.; Curionópolis (Equatorial Rainforest and “Campos Rupestres”), Iron Ore: Gruta SL 74CL, (MNRJ) 1 spec, 17–24.x.2008, Andrade, R.; **GOIÁS**: São Domingos (Cerrado), Limestone: Lapa São Bernardo II, (UFSCAR) 1 spec, 19.v.2015, Bichuette, ME.; Lapa São Vicente II, (UFSCAR) 1 spec, 06.iv.2016, Bichuette, ME.; Lapa Terra Ronca II, (UFSCAR) 1 spec, 01.x.2012, Gallão, JE.; Lapa Angélica, (UFSCAR) 1 spec, 29.iv.2011, Gallão, JE.; Lapa São Vicente II, (UFSCAR) 1 spec, 06.iv.2016, Bichuette, ME.; **BAHIA**: Paripiranga (Caatinga), Limestone: Abismo do Lixo, (UFSCAR) 1 spec, ix.2013, Rocha, KG.; Carinhanha (Caatinga), Limestone: Gruna da Altina, (UFSCAR) 5 spec, 27.xi.2015, Bichuette, ME.; **MINAS GERAIS**: Presidente Olegário (Cerrado and Semideciduous seasonal forest), Limestone: Lapa da Fazenda São Bernardo, (UFSCAR) 1 spec, in 30.xi.2013, Bichuette, ME., Zepon, T., Resende, LPA., Ribeiro, IA.; Monjolos (Cerrado), Limestone: Toca do Geraldo, (UFSCAR) 1 spec, 30.x.14, Fonseca-Ferreira, R.; Januária (Cerrado-Caatinga transition), Limestone: Gruta do Janelão, (UFSCAR) 2 spec, 22.vii.2012, Bichuette, ME.

######## Distribution.

Over 60 *Schendylops* species exist worldwide, but only 16 species have been found in Brazil, with none previously known to inhabit Brazilian caves. Most *Schendylops* species are from the Amazonia (Foddai et al. 2002) and Atlantic Rainforests in São Paulo, Rio de Janeiro, and Santa Catarina (Bücher 1940, 1942). Some species also occur in northeastern Brazil (Paraíba) (Bücher 1940, 1942). Here, we recorded *Schendylops* in iron ore and sandstone caves from Pará, as well as limestone caves from Goiás, Bahia, and Minas Gerais. Thus, the genus’ distribution was extended to central Brazil, Minas Gerais, and Bahia.

## Discussion

### Distribution of Brazilian cave centipedes

**Figure 3.**
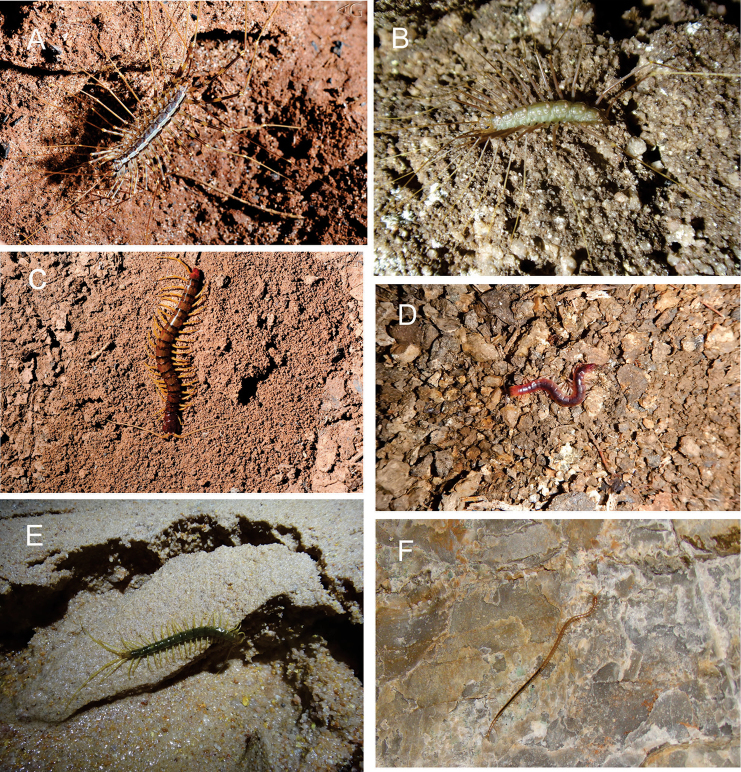
Centipedes from Brazilian caves *in loco*. **A** *Sphendononema guildingii* from limestone caves of Serra do Ramalho, Bahia state (A. Gambarini) **B** *Sphendononema* sp. from sandstone caves of Igatu, Chapada Diamantina, Bahia state (E.C. Igual) **C** *Scolopocryptops miersii* from limestone caves of Serra do Ramalho, Bahia state (A. Gambarini) **D** *Otostigmus tibialis* from granitic caves of Florianópolis, Santa Catarina state (M.E. Bichuette) **E** *Scolopocryptops troglocaudatus* from Igatu, Chapada Diamantina, Bahia state (E.C. Igual) **F** *Ribautia* sp. from a limestone cave of Peruaçu, Minas Gerais state (P.P. Rizzato).

The 563 centipede specimens recorded from 274 caves were assigned to four orders, ten families, 18 genera, and 47 morphospecies. Of the latter, 30 were identified to the species level, 12 to genus level, four to family level, and one to order level (Tables 1 and 2, Figure 3). Scolopendromorpha represents 41% of the centipede specimens, followed by Geophilomorpha (26%), Scutigeromorpha (23%), and Lithobiomorpha (10%) (Table 1). These records represent 21% of the Brazilian centipede fauna.

**Table 1.** Number of records of centipedes from Brazilian caves considering family, genera, species, and morphospecies.

Order	Family	Genera	Species	Morphospecies	Total specimens
Scutigeromorpha	2	2	2	2	128
Lithobiomorpha	1	1	–	1	59
Scolopendromorpha	3	9	28	33	231
Geophilomorpha	4	6	–	11	145
Total	10	18	30	47	563

The scutigeromorph centipedes were represented by *Sphendononema guildingii* and *Thereuoquima admirabilis*, respectively belonging to the Pselliodidae and Scutigeridae families. Scolopendridae, Cryptopidae, and Scolopocryptopidae represented the order Scolopendromorpha. The scolopendrid family was represented by six genera: *Cormocephalus*, *Rhoda*, *Scolopendropsis*, *Scolopendra*, *Otostigmus*, and *Rhysida*; cryptopids by only one genus (*Cryptops*); and the scolopocryptopids by two genera: *Scolopocryptops* and *Newportia*. The genera *Cormocephalus*, *Rhoda*, *Scolopendropsis*, *Scolopendra*, and *Rhysida* were each represented by one species. *Newportia*, *Otostigmus*, *Cryptops*, and *Scolopocryptops* were represented by nine, five, five, and four species, respectively. Therefore, *Newportia* is the most representative genus in Brazilian caves. Moreover, *Sphendononema*, *Cryptops*, *Scolopocryptops*, *Lamyctes*, *Newportia*, *Schendylops*, and *Otostigmus* genera were the most abundant centipedes collected in Brazilian caves, each with 31–123 individuals. Except *Ribautia* (16 specimens), other genera (*Thereuoquima*, *Cormocephalus*, *Rhoda*, *Scolopendropsis*, *Scolopendra*, *Rhysida*, *Itiphylus*, *Hyphydrophilus*, *Schizonampa*, and *Orphnaeus*) were less well represented, with fewer than six specimens each. *Sphendononema guildingii* is by far the most common species found in caves, followed by *Scolopocryptops miersii*, *Scolopocryptops troglocaudatus*, and *Newportia balzanii*. Owing to specimen issues (damage, lacking the ultimate pair of legs), the genus *Cryptops* had 54 unidentified specimens. However, the few well-preserved *Cryptops* specimens seem to represent seven unknown species that require further study.

### Distribution per lithology – sampling gaps represent distribution gaps

**Figure 4.**
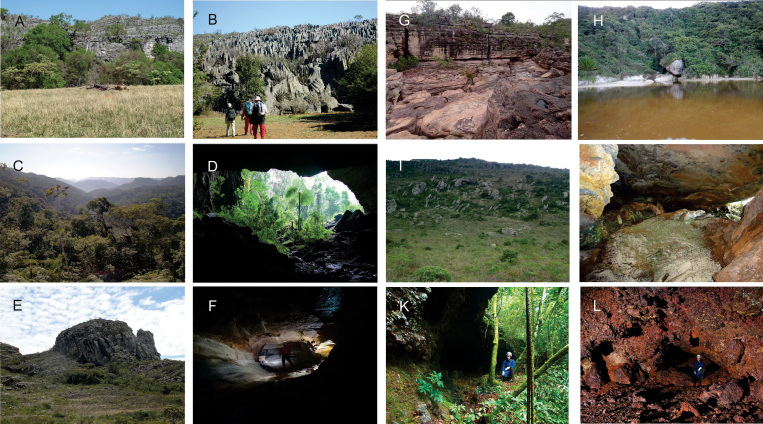
Outcrops and caves from Brazil with centipede records. **A** Limestone outcrops with pastures from Peruaçu region, Minas Gerais state. Cerrado (savannah-like vegetation) interspersed with Caatinga (xerophitic vegetation) (M.E. Bichuette) **B** Limestone outcrops with pastures from Serra do Ramalho, Bahia state. Caatinga (xerophitic vegetation) (M.E. Bichuette) **C** Atlantic Rainforest and limestone outcrops from Alto Ribeira, São Paulo state (M.E. Bichuette) **D** Alto Ribeira cave entrance, São Paulo state (M.E. Bichuette) **E** Quartizite outcrops with “Campo rupestre” vegetation from Diamantina, Minas Gerais state (M.E. Bichuette) **F** Sandstone cave from Igatu, Chapada Diamantina, Bahia state (E.C. Igual) **G** Sandstone outcrop from Igatu, Chapada Diamantina, Bahia state (J.E. Gallão) **H** Granite outcrop and Atlantic Rainforest from Florianópolis, Santa Catarina state (J.E. Gallão) **I** Iron ore outcrop with Cerrado from Caeté, Minas Gerais state (M.E. Bichuette) **J** Iron ore cave from Caeté, Minas Gerais state (M.E. Bichuette) **K, L** Iron ore caves and Canga vegetation from Canaã dos Carajás, Pará state (A. Coelho).

Cave centipedes are distributed in six different lithological types in Brazil (limestone, sandstone, quartzite, granite, iron ore, and shale). Limestone caves contained 43 % of the specimens (Figure 4a–d); iron ore caves contained 41 % (Figure 4i, j, k, l); sandstone caves, 10 % (Figures 4f and g); quartzite caves, 2 % (Figure 4e); granitic caves, 2 % (Figure 4h); and shale caves, 2 % (Table 2). Despite higher sampling effort due to historical projects that have continued and intensified the late 1970s, limestone caves did not possess more centipedes than iron ore caves. However, centipede distribution in limestone caves is significantly higher than in the remaining lithologies (sandstone, quartzite, granite, and shale).

**Table 2.** Distribution and number of records of centipedes from Brazilian caves per lithology. An asterisk (*) denotes species found exclusively in caves.

Taxon	Lithology						Total
	Limestone	Sandstone	Quartzite	Granite	Shale	Iron ore	
*Sphendononema guildingii*	40	6	1	–	–	76	123
*Thereuoquima admirabilis*	1	–	–	4	–	–	5
*Lamyctes* sp.	38	1	2	–	–	18	59
*Cormocephalus impressus*	–	1	–	–	–	–	1
*Rhoda thayeri*	–	–	–	–	–	1	1
*Scolopendropsis bahiensis*	–	–	1	–	–	–	1
*Scolopendropsis sp.*	–	–	–	–	–	1	1
*Scolopendra viridicornis*	5	–	–	–	–	–	5
*Otostigmus (Dactylotergitius) caudatus*	1	2	–	–	–	–	3
*Otostigmus (Parotostigmus) amazonae*	–	–	–	–	–	1	1
*Otostigmus (Parotostigmus) muticus*	4	–	–	–	–	–	4
*Otostigmus (Parotostigmus) tibialis*	5	–	2	–	–	–	7
*Otostigmus (Parotostigmus) tidius*	–	–	1	–	–	–	1
*Otostigmus* sp.	8	2	1	1	–	3	15
*Rhysida brasiliensis*	–	3	–	–	–	–	3
*Cryptops (Cryptops) spelaeoraptor	1	–	–	–	–	–	1
*Cryptops (Trigonocryptops) galatheae*	1	–	–	3	–	–	4
*Cryptops (Trigonocryptops) iheringi*	1	–	–	–	–	–	1
*Cryptops (Trigonocryptops) iporangensis	5	–	–	–	–	–	4
*Cryptops (Trigonocryptops) hephaestus	–	–	–	–	–	9	3
*Cryptops* sp.	32	7	2	–	–	14	61
*Scolopocryptops ferrugineus macrodon*	–	5	–	–	2	–	7
*Scolopocryptops melanostoma*	–	–	–	–	1	–	1
*Scolopocryptops miersii*	8	6	–	–	3	24	41
*Scolopocryptops troglocaudatus	–	12	–	–	–	–	12
*Newportia (Newportia) ernsti ernsti*	–	–	–	–	1	6	7
*Newportia (Newportia) ernsti fossulata*	–	–	–	–	–	3	3
*Newportia (Newportia) lasia*	–	–	–	–	–	2	2
*Newportia (Newportia) phoreta*	–	–	–	–	–	2	2
*Newportia (Newportia) potiguar	2	–	–	–	–	–	2
*Newportia (Newportia) spelaea	1	–	–	–	–	–	1
*Newportia (Tidops) balzanii*	11	–	–	–	–	–	11
*Newportia (Tidops) nisargani*	2	–	–	–	–	3	5
*Newportia (Tidops) simus*	–	1	–	–	–	–	1
*Newportia (Tidops*) sp.	3	6	–	–	–	8	17
*Newportia (Tidops*) sp.	–	–	–	–	–	2	2
Geophilomorpha fam. gen. sp.	16	–	–	–	–	6	22
Ballophilidae gen. sp.	5	–	–	–	–	7	12
*Ityphilus* sp.	1	–	–	–	–	2	3
Geophilidae gen. sp.	4	2	–	–	1	11	18
*Hyphydrophilus* sp.	–	–	–	–	–	3	3
*Ribautia* sp.	16	–	–	–	–	–	16
*Schizonampa* sp.	–	–	–	–	2	–	2
Oryiidae gen. sp.	1	–	–	–	–	–	1
*Orphnaeus* sp.	–	2	–	–	–	1	3
Schendylidae gen. sp.	17	–	–	1	1	3	22
*Schendylops* sp.	15	–	–	–	3	25	43
TOTAL	244	55	10	9	14	231	563

Some cave types remain neglected. Sandstone caves were eventually sampled during the 1980s and 1990s, but collection efforts only began in earnest during 2007–2009 (M.E. Bichuette pers. obs). Similarly, granitic caves were sampled in the 1980s and 1990s, but new field explorations only began in 2012 (continuing to date). Quartzite caves were likewise only explored starting from 2012.

**Figure 5.**
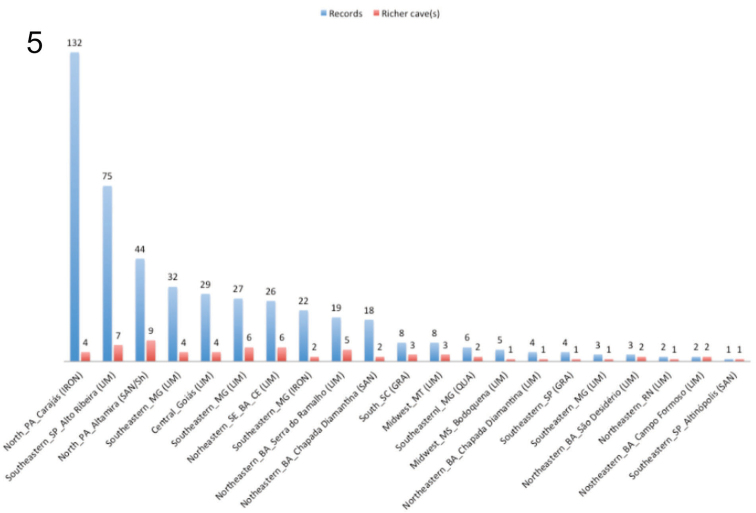
Total records per region and respectively richer caves. Lithologies: IRON, Iron ore; LIM, Limestone; SAN, Sandstone; Sh, Shale; GRA, Granitic; QUA, Quartzitic. States: PA, Pará; SP, São Paulo; MG, Minas Gerais; SE, Sergipe; BA, Bahia; CE, Ceará; SC, Santa Catarina; MT, Mato Grosso; MS, Mato Grosso do Sul; RN, Rio Grande do Norte.

The high number of records observed in iron ore caves comes from collections in the last 12 years; they are associated with environmental consulting firms that survey regions for potential iron exploitation, mainly in Minas Gerais and Pará. However, other cave lithologies, even in regions with small sampling effort, contained more species/morphospecies (Figure 5). For example, the cave with the most records was the Leonardo da Vinci shale cave (Pará), a poorly sampled lithology. This cave was explored in the 1980s (Trajano and Moreira 1991) and recently (2008 and 2009) by the MEB team, resulting in nine centipede species. In contrast, the Areias cave system from the Alto Ribeira karst area (São Paulo) is the best-studied cave in Brazil, with systematic collections since the 1980s, and was second in centipede species count (Figure 5). These results reinforce the fact that multiple factors should be considered when discussing distribution, rarity, and ecological patterns in cave fauna.

Such factors include replicated sampling effort, cave representativeness in terms of outcrops/massifs, paleoclimatic events, and biogeographical histories. The scutigeromorph *S. guildingii* and the lithobomorph *Lamyctes* spp. were recorded in most lithologies, except granite and shale. The scolopendromorphs of the genera *Otostigmus* and *Cryptops* were recorded in five lithologies, except shale. *Scolopocryptops* occurs in four lithologies (except quartzite and granite), with some species (e.g., *S. troglocaudatus*) preferring sandstone cave and others (*S. ferrugineus macrodon*) preferring sandstone or shale caves. Most *Newportia* species were found in only one type of lithology. *Newportia ernsti fossulata*, *N. lasia*, and *N. phoreta* were recorded in iron ore caves, while *N. potiguar*, *N. spelaea*, and *N. balzanii* were in limestone caves. *Newportia nisargani* was the only exception, recorded in both limestone and iron ore caves. Geophilomorph morphospecies were recorded in five lithologies, except quartzite. Genera *Hyphydrophilus* and *Ribautia* were only recorded in iron ore and limestone caves, respectively. The family Schendylidae is the most widespread geophilomorph group, occurring in four lithologies, except sandstone and quartzite.

Importantly, we only had partial access to materials collected in Brazilian caves, because some specimens (e.g., those collected for environmental licensing) are not deposited in official collections or lacked voucher numbers. Therefore, our current results should be considered preliminary.

### Troglobitic centipedes from Brazil and conservation considerations

Six Brazilian centipede species from *Cryptops*, *Newportia*, and *Scolopocryptops* were considered troglobitic by previous studies.

*Cryptops hephaestus* was recorded in three iron ore caves of Itabirito (Minas Gerais), two of them near each other, and the third farther away (Ázara and Ferreira 2013). Over 100 caves occur between these three caves and are poorly sampled. We examined 15 specimens from 12 caves of the Itabirito region; five were determined as *C. hephaestus*, but the remainder (juvenile or damaged individuals) were simply *Cryptops* sp. These new records suggest that *C. hephaestus* may occur outside caves, changing its categorization to troglophile. Alternatively, it may be a widely distributed troglobite.

*Cryptops iporangensis* was described on the basis of a single specimen from the Ressurgência das Areias de Água Quente cave, which is close to be not part of the Alto Ribeira Touristic State Park (PETAR), Iporanga, São Paulo. This species seemed to be rare in the region, with an occurrence range of <5,000 km^2^ (based on a map of the Areias cave system). Parts of the Areias caves are not within the protected state park. Thus, deforestation around the cave and unregulated tourist activity may negatively affect the cave itself. Because of these considerations, the species is currently categorized as Endanger. Our new records located this species in three other caves of PETAR, extending its distribution to other outcrops. We therefore suggest a review of *C. iporangensis* conservation status before the Red List of Brazilian Threatened Fauna is next updated.

*Cryptops spelaeoraptor* was recorded in the Caatinga phytophysiognomy, from a unique limestone cave of Bahia (Toca do Gonçalo). The species show marked troglomorphic traits, such as long trunk, antennae, and legs, as well as a high density of long setae on the cephalic plate plus the first three antennal articles (Ázara and Ferreira 2014a). The species also seems to be rare, because only one individual was collected after multiple several visits. The cave is located in an extremely dry site, and the species’ presence outside cave is very unlikely.

Two records of troglobitic species were obtained from *Newportia*. *Newportia spelaea* was found in Toca do Gonçalo (never outside) and presented marked troglomorphic traits. *Newportia potiguar* was described from multiple caves located in Rio Grande do Norte, suggesting a wider distribution than previously thought. Although Ázara and Ferreira (2014b) considered the two *Newportia* species as true troglobites, due to marked troglomorphic traits and exclusivity in cave habitats, some characters (e.g., long ultimate legs, pronounced depigmentation, and reduced cuticle sclerotization) are actually characteristic of *Newportia* juveniles. The *N. spelaea* and *N. potiguar* specimens could potentially be juveniles given their body lengths were 19 and 24 mm in body length, respectively. Therefore, the troglobitic status of both species must be reviewed. Additional collections, including epigean ones, are necessary for proper assignment of the species to an ecological-evolutionary category.

The fifth troglobitic centipede belongs to the genus *Scolopocryptops* and was recently described for sandstone caves from Bahia. *Scolopocryptops troglocaudatus* is the second troglobitic species of this genus to be found in Brazil and presents at least three robust troglomorphic characters: extremely long ultimate legs (exceeding 2/3 of the body length: 26.2 mm), long antennae, and reduced cuticle sclerotization (Chagas– Jr and Bichuette 2015). This species is known from 12 specimens distributed across four sandstone caves of Chapada Diamantina (Igatu region). Several well-sampled (including replicates) limestone caves are also nearby (Gallão and Bichuette 2015). However, since the beginning of sampling in 2006, no *S. troglocaudatus* specimens have been recorded inside or outside of these limestone caves. We therefore conclude that *S. troglocaudatus* is troglobitic species endemic to sandstone caves from the Igatu region.

Of the 15 myriapod species in the List of Brazilian Threatened Fauna, three are centipedes: *Scolopendropsis duplicata* Chagas-Jr, Edgecombe & Minelli 2008, *Cryptops spelaeoraptor*, and *Cryptops iporangensis* (the latter two both troglobitic) (MMA 2016). The first is categorized as Vulnerable and the remainder as Endanger.

## Supplementary Material

XML Treatment for Sphendononema guildingii

XML Treatment for Thereuoquima admirabilis

XML Treatment for Lamyctes

XML Treatment for Cormocephalus impressus

XML Treatment for Rhoda thayeri

XML Treatment for Scolopendropsis bahiensis

XML Treatment for Scolopendropsis

XML Treatment for Scolopendra viridicornis

XML Treatment for Otostigmus (Dactylotergitius) caudatus

XML Treatment for Otostigmus (Parotostigmus) amazonae

XML Treatment for Otostigmus (Parotostigmus) muticus

XML Treatment for Otostigmus (Parotostigmus) tibialis

XML Treatment for Otostigmus (Parotostigmus) tidius

XML Treatment for Otostigmus (Parotostigmus)

XML Treatment for Rhysida brasiliensis

XML Treatment for Cryptops (Cryptops) spelaeoraptor

XML Treatment for Cryptops (Trigonocryptops) galatheae

XML Treatment for Cryptops (Trigonocryptops) iheringi

XML Treatment for Cryptops (Trigonocryptops) iporangensis

XML Treatment for Cryptops (Trigonocryptops) hephaestus

XML Treatment for Cryptops

XML Treatment for Scolopocryptops ferrugineus macrodon

XML Treatment for Scolopocryptops melanostoma

XML Treatment for Scolopocryptops miersii

XML Treatment for Scolopocryptops troglocaudatus

XML Treatment for Newportia (Newportia) ernstiernsti

XML Treatment for Newportia (Newportia) ernstifossulata

XML Treatment for Newportia (Newportia) phoreta

XML Treatment for Newportia (Newportia) potiguar

XML Treatment for Newportia (Newportia) spelaea

XML Treatment for Newportia (Newportia)

XML Treatment for Newportia (Tidops) balzanii

XML Treatment for Newportia (Tidops) nisargani

XML Treatment for Newportia (Tidops) simus

XML Treatment for Newportia (Tidops)

XML Treatment for Geophilomorpha

XML Treatment for Ballophilidae

XML Treatment for Ityphilus

XML Treatment for Geophilidae

XML Treatment for Hyphydrophilus

XML Treatment for Ribautia

XML Treatment for Schizonampa

XML Treatment for Oryidae

XML Treatment for Orphnaeus

XML Treatment for Schendylidae

XML Treatment for Schendylops
